# Supplementary material for: Climate and ecology predict latitudinal trends in sexual selection inferred from avian mating systems
Source: PLoS Biol. 2024 Nov 4;22(11):e3002856. doi: 10.1371/journal.pbio.3002856 (PMC11567637; doi:10.1371/journal.pbio.3002856)
Supplement: S1 Text — Supplementary methods, results and discussion. The data and code can be found at https://doi.org/10.6084/m9.figshare.27255609. (DOCX) [file pbio.3002856.s001.docx]

**SUPPLEMENTARY INFORMATION**

**Climate and ecology predict latitudinal trends in sexual selection inferred from avian mating systems**

**Supplementary methods and results**

*Taxonomy and phylogeny*

To allow the use of phylogenetic analyses, we aligned taxonomy and nomenclature with the global BirdTree phylogeny [1]. Given the rapid state of flux in avian taxonomy, the species limits and family classifications in BirdTree data are increasingly obsolete, and the phylogenetic tree itself is likely to be replaced with new phylogenies using updated taxonomic treatments. To maximise future utility of our dataset, we rescored sexual selection and all other variables according to Clements *et al.* [2] taxonomy. This will allow integration of our dataset with ecological and life history data published in Birds of the World (https://www.birdsoftheworld.org/bow), as well as spatially explicit citizen-science data from eBird (https://www.ebird.org), and forthcoming global phylogenetic datasets [3].

Approximately 93% of species in the BirdTree dataset were directly matched with Clements species, including many that needed to be matched manually because of changes in spelling or higher taxonomic classification. In all these cases, we transferred the same sexual selection and data uncertainty scores from BirdTree to the Clements dataset. For the remaining 7% of species, 512 were taxonomic splits, where a BirdTree species was separated into multiple Clements species. In these cases, we identified the parent (nominate) species and transferred trait data directly from BirdTree to the parent lineage. For the remaining daughter species, we inferred sexual selection scores from the behaviour of the parent. For example, under BirdTree taxonomy, the House Sparrow *Passer domesticus* is considered a single species, with the Italian Sparrow considered a subspecies (*P. domesticus italiae*). However, under Clements taxonomy, *P. italiae* is considered a separate species. In this instance, we directly transferred sexual selection and data certainty scores from the BirdTree “*P. domesticus”* values to the Clements “*P. domesticus*” values. For *P. italiae*, we used the same sexual selection score as the parent by inference, because breeding behaviour of this geographically restricted form appears similar to the more widespread *P. domesticus*. Nonetheless, we dropped the data certainty score from 4 to 3 reflecting the reduction in certainty that arises because *P. italiae* occupies a small geographical range and the sexual selection score is no longer based on direct species-specific evidence.

The reverse procedure was required in cases where multiple BirdTree species have been merged into a single eBird species. These cases were less frequent, with only 219 BirdTree species combined into 102 eBird species. In almost all cases, sexual selection scores were the same across each set of BirdTree species, so this score was directly transferred to the corresponding eBird species into which they were merged. The only exception was the Ring-necked Pheasant (*Phasianus colchicus*), in which two BirdTree sister species were scored differently: the mainland nominate *P. colchicus* was scored 3 for polygamy, whereas the Green Pheasant (*P. versicolor*), a Japanese endemic, was scored 2 because a higher incidence of monogamy was reported than for mainland populations. Under eBird taxonomy, *P. colchicus* was merged with *P. versicolor* and was scored 3 because the Japanese form makes up only a small part of the total population.

When transferring data from BirdTree to eBird taxonomy, 31 species presented challenges because data certainty scores differed between daughter taxa being merged together (lumped) into a single parent taxon. In most of these problematic cases, the sexual selection score of widespread parent taxa was based on direct data from primary sources, whereas this direct data often only applied to one of the BirdTree daughter species (splits). In these instances, we transferred the certainty data from the nominate BirdTree species to the lumped (parent) eBird species. The only exception to this rule was *Stercorarius antarcticus* which is considered two separate species (*Catharacta lonnbergi* and *Catharacta antarctica*) by BirdTree and a single species by eBird. Under BirdTree, the daughter species or split, *C. lonnbergi*, carries a higher data certainty score of 4 because its sexual selection score is based on a molecular study of extra-pair paternity [4]. In the same treatment, *C. antarctica* has a lower certainty of 3, which is upgraded to 4 (higher certainty) under eBird taxonomy wherein *C. lonnbergi* is subsumed into *C. antarctica* (and transferred to the genus *Stercorarius*).

In five cases, BirdTree species were initially lumped and then subsequently re-split into multiple daughter species. However, sexual selection happened to be scored 0 (strict monogamy) in all these cases, simplifying the direct transfer of scores from BirdTree to eBird datasets. Finally, 176 eBird species had no corresponding BirdTree species. The majority of cases (*n* = 144 species) were extinct species that were omitted from BirdTree, which we did not include in our database. The remaining 32 species were described in the last decade, representing new additions to global species lists. In these cases, we scored sexual selection from literature following the same protocol used for BirdTree species (see Methods).

We include a taxonomic crosswalk (sheet 5) in S1 Data. This resource lists each BirdTree and eBird species aligned in neighbouring columns, showing how they are matched in our data transfer process. Specifically, we include the type of link between the two taxonomies (one-to-one, many-to-one, one-to-many, many-to-many) and highlight the parent taxon in cases where data are duplicated across multiple taxa in the case of splits. The crosswalk is designed to be used alongside the R script (clements_conversion.R) included with this study. As a disclaimer, our crosswalk may contain small errors as it was designed to transfer traits between thousands of species. We recommend that users carefully check the conversion of traits, paying particular attention to species which have transitioned between families or orders. We hope that the eBird dataset and associated taxonomic crosswalk can support future researchers by facilitating the transfer of traits to and from eBird taxonomy, including cases where lumped BirdTree species have conflicting data. This should be a useful resource for macroecological studies combining data from AVONET [5], Birds of the World, and eBird, or for researchers wishing to integrate their datasets with future phylogenies based on eBird taxonomy [3].

*Sexual selection metrics*

To assess the reliability of our scoring system, we collected data on three complementary and widely used measures of sexual selection from existing published datasets: residual testes mass [6,7], Bateman gradients (β_SS_) [8,9], and the opportunity for sexual selection (*I_S_*) [10]. Residual testes size constitutes a commonly used proxy for the strength of post-copulatory sexual selection on males whereas β_SS_ and *I_S_* are standardized metrics for the strength of pre-copulatory sexual selection. To increase our sample size, we also calculated metrics for additional species where possible, based on published data.

Residual testes mass refers to the mass of testes after accounting for total body mass. This metric provides information about the levels of sperm competition between rival males, which correlates with some aspects of sexual selection [11]. To calculate residual testes mass, we used recent data from Baker *et al.* [7] (*n* = 901 species), with an additional 76 species sourced from Pitcher *et al.* [6]. Because Baker *et al*. [7] published testes mass and body mass as separate measurements, we calculated residual testes mass under the same procedure as Pitcher *et al.* [6], using the residuals from the regression of log testes mass on log body mass.

*I_S_* can be calculated as the sex-specific variance in relativized mating success across individuals of a population (defined as the variance in the number of sexual partners per individual, divided by the squared mean of the number of sexual partners). We calculated *I_S_* for 28 unique species, either directly from information presented in papers, or from data subsequently provided by authors. Given the scarcity of studies that calculate quantitative measures of sexual selection, we also estimated *I_S_* for 51 monogamous species that recorded 0% extrapair paternity (EPP), indicating true genetic monogamy. Assuming that each individual was paired, and that operational sex-ratios are approximately even (as tends to be true for genetically monogamous species), this meant we could assign an *I_S_* of zero for these 51 additional species. As a sensitivity analysis, we removed these species from our reliability assessment (see main Methods), which produced near-identical results (Table A in S2 Text).

Bateman gradients (also termed sexual selection gradients) represent the slope of an ordinary least squares regression of reproductive success (number of offspring) on mating success (typically quantified as the number of mating partners) and therefore estimate the fitness benefit that can be obtained from additional matings. To compare between species, we converted Bateman gradients into standardised Pearson correlation coefficients. Lastly, because our sexual selection scores are bi-directional (scoring polyandry and polygyny equally), we took the recorded *I_S_* and β_SS_ from the sex with the highest value to generate a single measure per species. That is, the value for a particular species is from whichever sex experiences the most intense sexual selection.

*Phylogenetic signal*

We tested the phylogenetic signal of sexual selection in birds using a method devised for binary traits (*D*) [12]. Thus, we dichotomized sexual selection by grouping scores as either low sexual selection (0–2) or high (3–4). Results show that phylogenetic *D* was indistinguishable from zero (*D* = -0.19, *p* = 0.98), suggesting that sexual selection strength is highly conserved in birds and has likely evolved under a Brownian motion model.

*Spatial methods*

To understand how sexual selection varies globally, we incorporated spatial information using expert-drawn geographical range maps provided by Birdlife International [13]. To align species with BirdTree [1], we used the BirdLife-to-BirdTree taxonomic crosswalk provided in AVONET [5]. In most cases, BirdTree species had a single equivalent BirdLife species name (one-to-one matches; *n* = 8949), which allowed us to use the corresponding geographical range polygon for the same species.

The majority of taxonomic differences between BirdTree and BirdLife were splits, which resulted in a single BirdTree species corresponding with multiple BirdLife species (BirdLife splits; *n* = 1929). In these cases, we combined multiple BirdLife daughter species into a single range polygon for their corresponding parent BirdTree species using the *sf* package [14]. In the small number of cases where multiple BirdTree species correspond with one BirdLife species (BirdLife lumps; *n* = 198), we used BirdLife version 2.0 maps [13], created prior to any taxonomic changes. In this resource, we used maps for the corresponding BirdLife synonyms for each BirdTree species.

For a small number of BirdLife lumps (*n* = 64), no previous geographical range polygon existed as a version 2.0 map. In addition, some taxonomically stable species lacked geographical data (*n* = 57), either because no part of their known range is coded as resident or breeding range, or because the map is not openly released by BirdLife International (typically because the species is sensitive to hunting or trapping for the cage-bird trade). For mapping purposes, we restricted range polygons to areas where the species is coded by BirdLife International as extant and either native or reintroduced. Finally, because latitude and environmental conditions in the non-breeding range are largely irrelevant to levels of sexual selection, we limited our selection to the breeding and resident ranges of each study species.

To extract species-specific data, we used a Behrmann equal-area projection for geographical range polygons. To calculate a single value of latitude for each species, we determined the geometric centroid of each range polygon using the *PBSmapping* package [15]. This approach was preferred over midpoint latitude metrics because the centroid considers the overall shape of a species’ distribution, particularly important for wide-ranging species. Assigning a single value of latitude to each species greatly over-simplifies their distribution, and can be misleading, particularly in species with wide ranges spanning the equator. Nonetheless, midpoint or centroid estimates have the advantage of simplicity, removing the statistical problem of repeated measures per taxon, and usually provide a reasonable approximation of the latitude at which most individuals in the global population breed. For these reasons, midpoints or centroids are often used in phylogenetic comparative analyses or macroevolutionary models testing for latitudinal effects [16–18].

To assess global patterns of sexual selection, we first created a unified map by combining species geographical ranges into a single raster by overlapping the range with a grid of cells at 200-km resolution (Behrmann projection). To calculate average values of sexual selection per cell, we summed the sexual selection scores of all species with geographical ranges overlapping more than 50% of that cell, then divided the total by the corresponding species richness of the cell. This produced a single raster with cell values representing average sexual selection for the species present. We repeated this process using data certainty scores to generate a map of average data certainty for all birds and for each ecological partition listed in Table B in S2 Text. For visualisation purposes, we used the same procedure to create maps at a 5-km resolution.

To determine if sexual selection strength followed a latitudinal gradient, we modelled mean sexual selection across each 200-km grid cell using spatial simultaneous auto-regression (SAR) models from the R package *spatialreg* [19]. We incorporated spatial autocorrelation using a queen-based distance-neighbourhood matrix weighted by the standardised sum of all links (*W*), as this weighting style produced the lowest Akaike information criterion values in subsequent models. Each SAR model used absolute latitude (distance from the equator) as the sole predictor, calculated from the centre of each grid cell. We first ran a single model predicting sexual selection strength for all species (*n* = 9836), which showed a strong positive gradient in sexual selection (Table B in S2 Text). Given the strong positive effect of latitude on sexual selection across species, we ran subsequent models to determine if the overall pattern was true for different ecological groups, using the same response and predictor. We then repeated each model using a conservative dataset (certainty score 3–4), which produced similar results.

*Bayesian ordinal models*

Bayesian mixed-effect models were constructed using the brms package in R [20]. Because our sexual selection scores are rankings, we used ordinal regression models with a cumulative family distribution. This model estimates the cumulative odds of being at or below each category level, providing insights into how the independent variables influence the likelihood of moving up or down the ordinal scale. Coefficient estimates and confidence intervals can be interpreted similarly to other standard generalised-linear models. Posterior predictive checks confirmed that a cumulative family distribution fits the data extremely well.

To further investigate latitudinal gradients in sexual selection strength identified during grid-cell based analyses, we modelled sexual selection score at the species level, with sexual selection as the response and absolute latitude as the predictor. We chose not to apply any additional transformations to latitude because models fitted well, and produced patterns that are easier to interpret and to compare with the adjoining maps. We then repeated the same analyses for each ecological group, which generally support the results of corresponding SAR models. The only exception was that we identified a negative latitudinal gradient in sexual selection strength for non-territorial species. This difference in results is presumably related to the much denser sampling of species in the tropics, which inflates the strength of the species-level analysis but is smoothed out in the SAR analysis where data are average within grid cells, making species richness irrelevant. To determine if species-level models were robust to data certainty, we additionally repeated each species-level model using a conservative dataset (certainty score 3–4), which produced consistent results to the full dataset.

For univariate and multivariate trait models, designed to identify the evolutionary determinants of sexual selection strength, we included a phylogenetic covariance matrix as a random effect, using the Jetz *et al.* [1] tree topology grafted to the Prum *et al*. [21] genomic backbone. Temperature seasonality – the only continuous variable – was log-transformed to approximate normality and standardised to two standard deviations to facilitate comparison with categorical predictors [22]. All categorical predictors were centred to reduce collinearity with interaction terms, and to set the reference level for each trait to the global average [23]. The variance inflation factors (VIF) of all model terms were below three, suggesting that collinearity between predictors does not affect model interpretation (Table C in S2 Text). To further address issues of collinearity, multivariate models used additional QR decomposition (whereby a matrix is expressed as the product of two separate matrices, Q and R). This approach helps recover accurate coefficient estimates in the presence of correlated traits [20].

For all Bayesian ordinal models, we used a no u-turn sampler (NUTS) and selected 10,000 total iterations with a 5,000-iteration warmup-phase and thinning every 20 iterations. Each model was run with two chains which we assessed for convergence by visually inspecting the mixing of chains and ensuring that r-hat values reached a value of one. To ensure our results are robust to uncertainty in tree topology, we repeated each model including phylogeny over 50 randomly selected trees, combining draws into a single posterior distribution. Following recommendations in Gelman [24], we assigned weakly informative priors to the intercept and slope parameters: normal (0,1). To improve sampling speed, and because initial models showed the estimate for the phylogenetic effect was consistently close to 1, we assigned a stronger prior to the phylogenetic effect: gamma (2,1). To assess the proportion of variance explained by model predictors, we used approximate *R^2^* values for Bayesian methods [25], adapted to fit ordinal regression models [26]. To calculate reported p-values from Bayesian regression models, we extracted the probability of direction from posterior draws, as described by Makowski *et al.* [27].

The R code used to run all analyses and prepare figures is available at https://github.com/ra-barber/sexual_selection

**Supplementary discussion**

Information about operational sex ratio (OSR) and parental care roles can be used as evidence for the strength of sexual selection. For example, a skewed sex ratio can lead to higher rates of intrasexual competition for mates in the majority sex, and greater mate selection in the minority sex, thus reflecting key elements of sexual selection [10]. Conversely, costly parental care is often cited as a major hypothesis explaining monogamy [28,29] because time constraints can prevent individuals from seeking additional mating opportunities. However, we excluded both OSR and parental care as components of sexual selection scores and predictors in our models for two main reasons. First, combining mating behaviour, OSR, and parental care as a single metric would require assigning thresholds to each trait at which sexual selection was approximately equal, while in reality, many polygamous and bi-parental species have similar OSRs regardless of sexual selection. Therefore, our decision to omit OSR as a direct contributor to our metric reflects a deliberate effort to prioritize the inclusion of mating behaviour, which although imperfect, may better align with sexual selection across diverse species. Second, suitable data is lacking for almost all species, so we are not able to include this information in a systematic way until more research is conducted on avian systems worldwide. In addition, some binary traits with better coverage such as developmental mode are unsuitable for our analyses because large sections of the tree have no variation, limiting their accuracy in phylogenetic models.

**Supplementary references**

1. Jetz W, Thomas GH, Joy JB, Hartmann K, Mooers AO. The global diversity of birds in space and time. Nature. 2012;491: 444–448. doi:10.1038/nature11631

2. Clements JF, Schulenberg TS, Iliff MJ, Billerman SM, Fredericks TA, Gerbracht JA, et al. The eBird/Clements checklist of birds of the world: v2021. 2021. Available: https://www.birds.cornell.edu/clementschecklist/download/

3. McTavish EJ, Gerbracht JA, Holder MT, Iliff MJ, Lepage D, Rasmussen P, et al. A complete and dynamic tree of birds. bioRxiv; 2024. p. 2024.05.20.595017. doi:10.1101/2024.05.20.595017

4. Millar C. Patterns of reproductive success determined by DNA fingerprinting in a communally breeding oceanic bird. Biol J Linn Soc. 1994;52: 31–48.

5. Tobias JA, Sheard C, Pigot AL, Devenish AJM, Yang J, Sayol F, et al. AVONET: morphological, ecological and geographical data for all birds. Ecol Lett. 2022;25: 581–597. doi:10.1111/ele.13898

6. Pitcher TE, Dunn PO, Whittingham LA. Sperm competition and the evolution of testes size in birds. J Evol Biol. 2005;18: 557–567. doi:10.1111/j.1420-9101.2004.00874.x

7. Baker J, Humphries S, Ferguson-Gow H, Meade A, Venditti C. Rapid decreases in relative testes mass among monogamous birds but not in other vertebrates. Ecol Lett. 2020;23: 283–292. doi:10.1111/ele.13431

8. Janicke T, Häderer IK, Lajeunesse MJ, Anthes N. Darwinian sex roles confirmed across the animal kingdom. Sci Adv. 2016;2: e1500983. doi:10.1126/sciadv.1500983

9. Fromonteil S, Marie-Orleach L, Winkler L, Janicke T. Sexual selection in females and the evolution of polyandry. PLoS Biol. 2023;21: e3001916. doi:10.1371/journal.pbio.3001916

10. Janicke T, Morrow EH. Operational sex ratio predicts the opportunity and direction of sexual selection across animals. Ecol Lett. 2018;21: 384–391. doi:10.1111/ele.12907

11. Lüpold S, de Boer RA, Evans JP, Tomkins JL, Fitzpatrick JL. How sperm competition shapes the evolution of testes and sperm: a meta-analysis. Philos Trans R Soc. B. 2020;375: 20200064. doi:10.1098/rstb.2020.0064

12. Fritz SA, Purvis A. Selectivity in mammalian extinction risk and threat types: a new measure of phylogenetic signal strength in binary traits. Conserv Biol. 2010;24: 1042–1051. doi:10.1111/j.1523-1739.2010.01455.x

13. BirdLife International. BirdLife Data Zone. 2021. Available: http://www.birdlife.org/datazone

14. Pebesma E. Simple features for R: standardized support for spatial vector data. R J. 2018;10: 439–446.

15. Schnute JT, Boers N, Haigh R. PBSmapping: mapping fisheries data and spatial analysis tools. R package version 2.70.3. 2017. doi:10.32614/CRAN.package.PBSmapping

16. Weir JT, Schluter D. The latitudinal gradient in recent speciation and extinction rates of birds and mammals. Science. 2007;315: 1574–1576. doi:10.1126/science.1135590

17. Sheard C, Neate-Clegg MHC, Alioravainen N, Jones SEI, Vincent C, MacGregor HEA, et al. Ecological drivers of global gradients in avian dispersal inferred from wing morphology. Nat Commun. 2020;11: 2463. doi:10.1038/s41467-020-16313-6

18. Weeks BC, Naeem S, Lasky JR, Tobias JA. Diversity and extinction risk are inversely related at a global scale. Ecol Lett. 2022;25: 697–707. doi:10.1111/ele.13860

19. Bivand R, Millo G, Piras G. A review of software for spatial econometrics in R. Mathematics. 2021;9: 1276. doi:https://doi.org/10.3390/math9111276

20. Bürkner PC. Brms: an R package for Bayesian multilevel models using stan. J Stat Softw. 2017;80: 1–28. doi:10.18637/jss.v080.i01

21. Prum RO, Berv JS, Dornburg A, Field DJ, Townsend JP, Lemmon EM, et al. A comprehensive phylogeny of birds (Aves) using targeted next-generation DNA sequencing. Nature. 2015;526: 569–573. doi:10.1038/nature15697

22. Gelman A. Scaling regression inputs by dividing by two standard deviations. Stat Med. 2008;27: 2865–2873. doi:10.1002/sim.3107

23. Schielzeth H. Simple means to improve the interpretability of regression coefficients. Methods Ecol Evol. 2010;1: 103–113. doi:10.1111/j.2041-210X.2010.00012.x

24. Gelman A. Prior distributions for variance parameters in hierarchical models. Bayesian Anal. 2006;1: 515–534.

25. Gelman A, Goodrich B, Gabry J, Vehtari A. R-squared for Bayesian regression models. Am Stat. 2019;73: 307–309. doi:10.1080/00031305.2018.1549100

26. McKelvey RD, Zavoina W. A statistical model for the analysis of ordinal level dependent variables. J Math Sociol. 1975;4: 103–120. doi:10.1080/0022250X.1975.9989847

27. Makowski D, Ben-Shachar MS, Chen SHA, Lüdecke D. Indices of effect existence and significance in the Bayesian framework. Front Psychol. 2019;10. doi:10.3389/fpsyg.2019.02767

28. Klug H. Why monogamy? A review of potential ultimate drivers. Front Ecol Evol. 2018;6. doi:10.3389/fevo.2018.00030

29. Kvarnemo C. Why do some animals mate with one partner rather than many? A review of causes and consequences of monogamy. Biol Rev. 2018;93: 1795–1812. doi:10.1111/brv.12421
