# Supplementary material for: Climate and ecology predict latitudinal trends in sexual selection inferred from avian mating systems
Source: PLoS Biol. 2024 Nov 4;22(11):e3002856. doi: 10.1371/journal.pbio.3002856 (PMC11567637; doi:10.1371/journal.pbio.3002856)
Supplement: S2 Text — Table A in S2 Text. Scoring system for assigning data certainty to estimates of sexual selection in birds. Table B in S2 Text. Comparing alternative metrics of sexual selection. Table C in S2 Text. Latitudinal gradients in sexual selection. Table D in S2 Text. Species-level latitudinal gradients in sexual selection. Table E in S2 Text. Ecological predictors of sexual selection. Table F in S2 Text. Results of sensitivity analyses assessing robustness of models to data certainty. Table G in S2 Text. Collinearity between multivariate model predictors. (DOCX) [file pbio.3002856.s002.docx]

**Table A.** **Scoring system for assigning** **data certainty to estimates of sexual selection in birds**. Bird species vary from well-known to almost entirely unknown in life, creating potential biases in knowledge. To account for this, we applied a classification system to quantify the availability of information relevant to the scoring procedure for estimating levels of sexual selection in each species. This classification system is adapted from Tobias *et al.* [1].

| **Score** | **Criteria** |  |
| --- | --- | --- |
| 1 | No direct or indirect evidence, including a lack of clear information preventing inference from closely related species. This score is mainly restricted to extremely rare species with no close relative, or poorly known species occurring in taxonomic groups with no clear pattern in mating systems. |  |
| 2 | Low levels of certainty due to conflicting evidence. This includes cases where inference is complicated by heterogeneity of sexual selection scores among closely related species (often caused by high levels of variation in mating systems across a clade) or where published evidence about mating systems is contradictory. |  |
| 3 | Moderate levels of certainty based on inference and reasonable circumstantial evidence. This includes reliable field observations suggestive of particular mating systems, as well as inference from closely related species. |  |
| 4 | High levels of certainty based on direct evidence published in primary and secondary literature. This includes both genetic evidence (EPP) and clear-cut behavioural observations, such as active leks or multi-year partnerships between individuals in long-lived socially monogamous species with stable pair-bonds. |  |

**Table B.** **Comparing alternative metrics of sexual selection.** Results shown are β estimates and 95% credible intervals (CI) from phylogenetic Bayesian ordinal mixed-effect models estimating the strength of relationship between sexual selection scores from this study and alternative measures of sexual selection: residual testes mass (a proxy for post-copulatory sexual selection), Bateman gradients (β_SS_; reproductive benefit from additional matings), the opportunity for sexual selection (*I_S_*; variance in mating success). To compare sex-specific metrics with bi-directional scores, we selected the largest β_SS_ or *I*_S_ from each species, treating males and female metrics equally. For 51 monogamous species with 0% extra-pair paternity reported in well-sampled molecular studies, we scored *I_S_* as zero. When we ran a sensitivity analysis (*I_S_*2) with these arbitrary zeroes excluded, results remained similar. Models were run on a sample of 50 phylogenetic tree topologies extracted from www.birdtree.org [2], grafted to the Prum *et al.* [3] genomic backbone. Testes mass = residual testes mass. Conditional *R^2^* = the total variation explained by each model. Marginal *R^2^* = the variation explained solely by sexual selection score, separate from phylogenetic effects. See S1 Text for detailed model descriptions. The data underlying this table can be found at https://doi.org/10.5281/zenodo.13625374.

| **Metric** | ***n*** | **Estimate** | **95% CI** | **Conditional *R*^2^** | **Marginal *R*^2^** |
| --- | --- | --- | --- | --- | --- |
| **Testes mass** | 977 | 2.41 | 1.69, 3.36 | 0.91 | 0.30 |
| **β_SS_** | 14 | 4.51 | 0.55, 10.86 | 0.76 | 0.53 |
| ***I_S_*** | 79 | 24.08 | 15.48, 37.94 | 0.98 | 0.97 |
| ***I_S_*2** | 28 | 24.24 | 15.59, 38.47 | 0.98 | 0.97 |

**Table C.** **Latitudinal gradients in sexual selection.** Results shown are β estimates and 95% confidence intervals (CI) from spatial simultaneous autoregression models estimating the strength of latitudinal gradients in sexual selection across different ecological groups of birds. Sample sizes refer to the number of species in each group. Models were repeated on a subset of species with higher-quality data (scored 3 or 4 for data certainty; Table A in S2 Text). Detailed model descriptions are provided in S1 Text; see Figs 3-4 and Figs S5-6 for visualisation. 1^ry^ consumer, Primary consumer; 2^ry^ consumer, Secondary consumer. The data underlying this table can be found at https://doi.org/10.5281/zenodo.13625374.

| **Sample** | **Certainty 1-4** | | | **Certainty 3-4** | | |
| --- | --- | --- | --- | --- | --- | --- |
|  | *n* | Estimate | 95% CI | *n* | Estimate | 95% CI |
| **All birds** | 9836 | 0.63 | 0.53, 0.75 | 7592 | 0.63 | 0.51, 0.74 |
| **1^ry^ consumers** | 2753 | -0.01 | -0.08, 0.23 | 2311 | -0.01 | -0.17, 0.15 |
| **Frugivores** | 1025 | -0.20 | -0.48, -0.10 | 767 | -0.20 | -0.39, -0.00 |
| **2^ry^ consumers** | 7083 | 0.74 | 0.64, 0.81 | 5281 | 0.74 | 0.66, 0.83 |
| **Invertivores** | 4694 | 0.94 | 0.78, 1.01 | 3477 | 0.94 | 0.84, 1.05 |
| **Migrants** | 901 | 0.49 | 0.19, 0.46 | 793 | 0.49 | 0.36, 0.62 |
| **Non-migrants** | 8935 | 0.69 | 0.61, 0.85 | 6799 | 0.69 | 0.57, 0.82 |
| **Territorial** | 7261 | 0.78 | 0.72, 0.90 | 5353 | 0.78 | 0.69, 0.88 |
| **Non-territorial** | 2575 | 0.08 | -0.15, 0.15 | 2239 | 0.08 | -0.07, 0.23 |

**Table D. Species-level latitudinal gradients in sexual selection.** Results shown are β estimates and 95% credible intervals (CI) from Bayesian ordinal mixed-effect models estimating the strength of latitudinal gradients in sexual selection across different ecological groups of birds. Models were run on all species, then repeated on a subset of species with higher-quality data (scored 3-4 for data certainty; Table A in S2 Text). To improve clarity, estimates were multiplied by 10 to represent the change in sexual selection score over a 10 degree increase in latitude. See S1 Text for details of models and Fig S7 for visualisation of results. 1^ry^ consumer, Primary consumer; 2^ry^ consumer, Secondary consumer. The data underlying this table can be found at https://doi.org/10.5281/zenodo.13625374.

| **Sample** | **Certainty 1-4** | | | **Certainty 3-4** | | |
| --- | --- | --- | --- | --- | --- | --- |
|  | *n* | Estimate | 95% CI | *n* | Estimate | 95% CI |
| **All birds** | 9836 | 0.17 | 0.14, 0.20 | 7592 | 0.15 | 0.12, 0.18 |
| **1^ry^ consumers** | 2753 | 0.01 | -0.04, 0.07 | 2311 | -0.03 | -0.09, 0.03 |
| **Frugivores** | 1025 | -0.24 | -0.47, -0.01 | 767 | -0.30 | -0.57, -0.05 |
| **2^ry^ consumers** | 7083 | 0.30 | 0.26, 0.34 | 5281 | 0.32 | 0.28, 0.36 |
| **Invertivores** | 4694 | 0.45 | 0.39, 0.51 | 3477 | 0.56 | 0.50, 0.63 |
| **Migrants** | 901 | 0.15 | 0.06, 0.23 | 793 | 0.14 | 0.05, 0.23 |
| **Non-migrants** | 8935 | 0.13 | 0.09, 0.17 | 6799 | 0.11 | 0.06, 0.15 |
| **Territorial** | 7261 | 0.42 | 0.37, 0.46 | 5353 | 0.42 | 0.37, 0.47 |
| **Non-territorial** | 2575 | -0.11 | -0.15, -0.06 | 2239 | -0.12 | -0.17, -0.08 |

**Table E.** **Ecological predictors of sexual selection.** Results shown are β estimates and 95% credible intervals (CI) from univariate and multivariate phylogenetic Bayesian ordinal mixed-effect models testing drivers of sexual selection in birds (*n* = 9836 species). Models were run on a sample of 50 phylogenetic trees extracted from www.birdtree.org [2], grafted to the Prum *et al.* [3] genomic backbone. See S1 Text for detailed model descriptions. See Figs 5 and 6 for visualisation. 1^ry^ consumer = primary consumer. For univariate models, the variance in sexual selection explained by each predictor is reported as the marginal *R*^2^, only considering fixed effects. The variance explained by combined predictors in the multivariate model was greater than all individual predictors (marginal *R*^2^ = 0.45). The data underlying this table can be found at https://doi.org/10.5281/zenodo.13625374.

| **Predictor** | **Univariate** | | | **Multivariate** | |
| --- | --- | --- | --- | --- | --- |
|  | Estimate | 95% CI | *R*^2^ | Estimate | 95% CI |
| **1^ry^ consumer** | 0.52 | 0.13, 0.92 | 0.02 | 0.64 | 0.21, 1.07 |
| **Migration** | 2.06 | 1.70, 2.43 | 0.12 | 0.84 | 0.44, 1.25 |
| **Territoriality** | -2.61 | -3.13, -2.12 | 0.34 | -2.71 | -3.27, -2.17 |
| **Seasonality** | 2.12 | 1.83, 2.43 | 0.31 | 1.78 | 1.44, 2.12 |
| **1^ry^ consumer × territoriality** | - | - |  | 0.80 | 0.08, 1.51 |
| **1^ry^ consumer × seasonality** | - | - |  | -0.75 | -1.33, -0.18 |

**Table F.** **Results of sensitivity analyses assessing robustness of models to data certainty.** Results shown are β estimates and 95% credible intervals (CI) from univariate and multivariate phylogenetic Bayesian ordinal mixed-effect models assessing potential drivers of sexual selection in birds, restricted to a subset of species with higher-quality data (scored 3 or 4 for data certainty; *n* = 7592 species). Models were run on a sample of 50 phylogenetic trees extracted from www.birdtree.org [2], grafted to the Prum *et al.* [3] genomic backbone. See S1 Text for detailed model descriptions. For univariate models, the variance in sexual selection explained by each predictor is reported as the marginal *R*^2^, only considering fixed effects. The variance explained by combined predictors in the multivariate model was greater than all individual predictors (marginal *R*^2^ = 0.54). 1^ry^ consumer, Primary consumer. The data underlying this table can be found at https://doi.org/10.5281/zenodo.13625374.

| **Predictor** | **Univariate** | | | **Multivariate** | |
| --- | --- | --- | --- | --- | --- |
|  | Estimate | 95% CI | *R*^2^ | Estimate | 95% CI |
| **1^ry^ consumer** | 0.48 | -0.01, 0.98 | 0.03 | 0.62 | 0.10, 1.16 |
| **Migration** | 1.87 | 1.46, 2.31 | 0.11 | 0.50 | 0.03, 0.96 |
| **Territoriality** | -3.03 | -3.66, -2.43 | 0.46 | -3.17 | -3.83, -2.55 |
| **Seasonality** | 2.45 | 2.04, 2.90 | 0.38 | 2.11 | 1.68, 2.58 |
| **1^ry^ consumer × territoriality** | - | - |  | 0.74 | -0.12, 1.59 |
| **1^ry^ consumer × seasonality** | - | - |  | -1.14 | -1.85, -0.44 |

**Table G**. **Collinearity between multivariate model predictors.** Results shown are variance inflation factors (VIF) calculated for multivariate models predicting sexual selection in birds. Columns show results for all birds (*n* = 9836 species) and a subset of species with high certainty data (*n* = 7592 species). High certainty data was defined as species scored 3 or 4 for data certainty (see Table A in S2 Text). All VIF were below 3, suggesting that collinearity should not impact model interpretation. 1^ry^ consumer, Primary consumer. The data underlying this table can be found at https://doi.org/10.5281/zenodo.13625374.

| **Predictor** | **Certainty 1-4** | **Certainty 3-4** |
| --- | --- | --- |
| **1^ry^ consumer** | 1.21 | 1.23 |
| **Migration** | 1.32 | 1.35 |
| **Territoriality** | 1.24 | 1.24 |
| **Seasonality** | 1.27 | 1.30 |
| **1^ry^ consumer × territoriality** | 1.20 | 1.17 |
| **1^ry^ consumer × seasonality** | 1.05 | 1.06 |

**Supplementary references**

1. Tobias JA, Sheard C, Seddon N, Meade A, Cotton AJ, Nakagawa S. Territoriality, social bonds, and the evolution of communal signaling in birds. Front Ecol Evol. 2016;4: 74. doi:10.3389/fevo.2016.00074

2. Jetz W, Thomas GH, Joy JB, Hartmann K, Mooers AO. The global diversity of birds in space and time. Nature. 2012;491: 444–448. doi:10.1038/nature11631

3. Prum RO, Berv JS, Dornburg A, Field DJ, Townsend JP, Lemmon EM, et al. A comprehensive phylogeny of birds (Aves) using targeted next-generation DNA sequencing. Nature. 2015;526: 569–573. doi:10.1038/nature15697
