# Supplementary figures and images for: Climate and ecology predict latitudinal trends in sexual selection inferred from avian mating systems
Source: PLoS Biol. 2024 Nov 4;22(11):e3002856. doi: 10.1371/journal.pbio.3002856 (PMC11567637; doi:10.1371/journal.pbio.3002856)

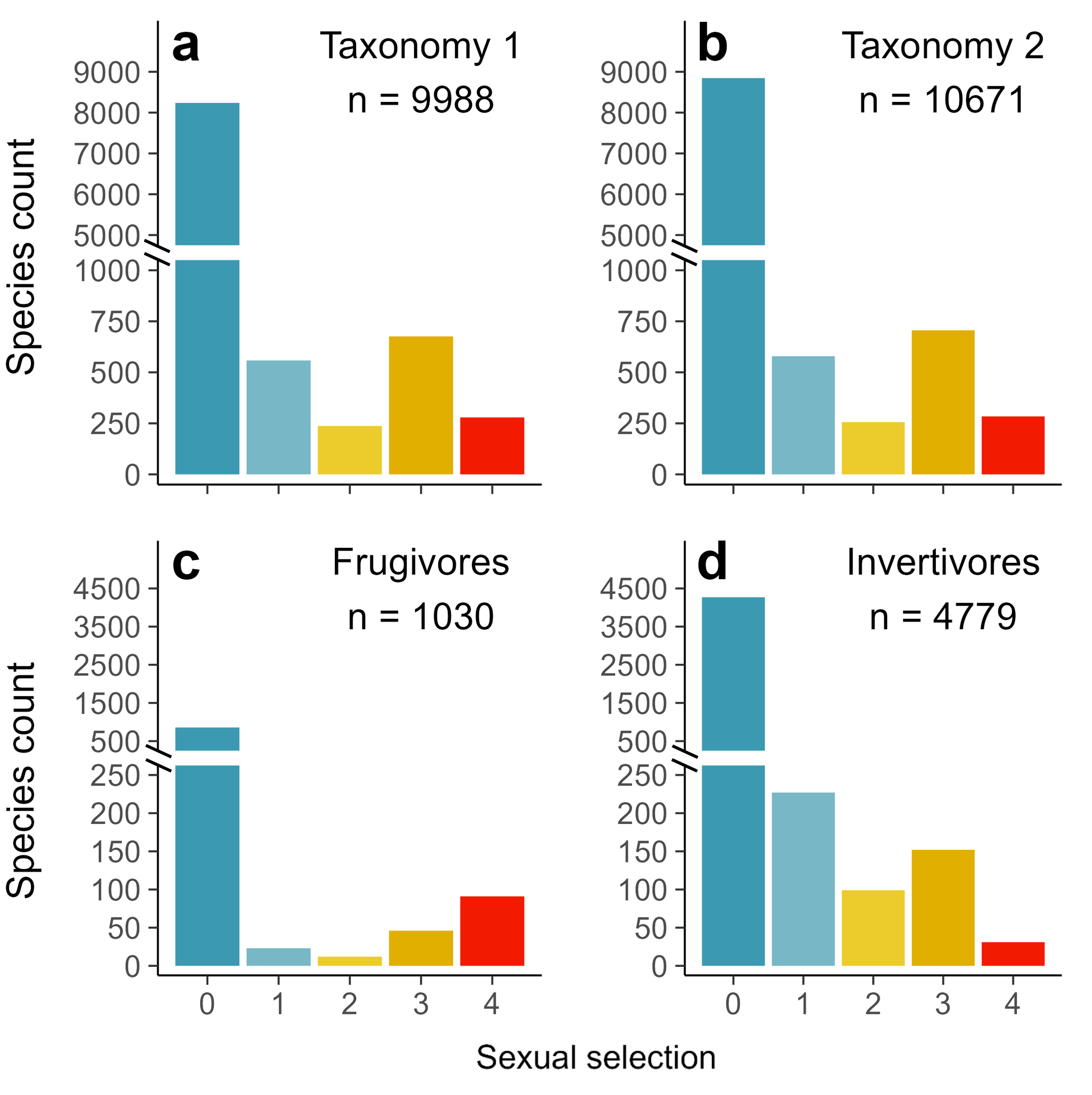

Supplement: S1 Fig — Plots show number of species in categories 0–4 according to the species limits in BirdTree [50] (a) or a more recent taxonomic update [57] (b), as well as within the 2 largest dietary guilds: invertivory (c) and frugivory (d). The strength of sexual selection increases from 0 (strict monogamy) to 4 (extreme polygamy) according to our scoring system (Table 1). The higher species total in Clements is caused by the addition of a few newly described species along with several hundred taxonomic splits. The data underlying this figure can be found at https://doi.org/10.6084/m9.figshare.27255609. (TIF) [file pbio.3002856.s003.tif]

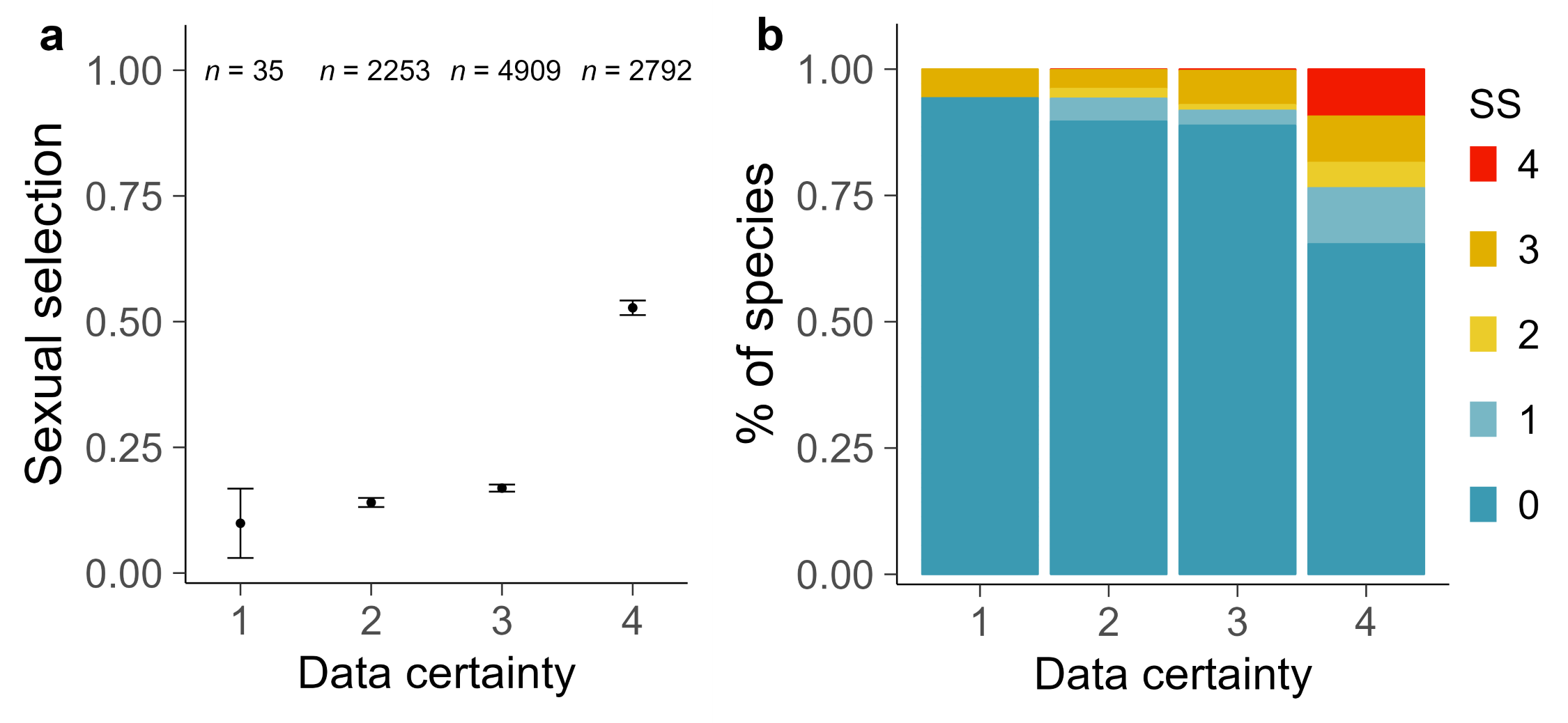

Supplement: S2 Fig — Plots show how the certainty in data for each species affects (a) average sexual selection and (b) variation in sexual selection score. In (a) and (b), sexual selection is scored from 0 (strict monogamy) to 4 (extreme polygamy; see Table 1) and data certainty is scored from 1 (no direct or indirect evidence) to 4 (direct evidence published in primary and secondary literature; see Table A in S2 Text). In (a), points show average sexual selection for each category of data certainty; whiskers denote one standard error; sample sizes are the total number of species in each data certainty partition. In (b), stacked bars show the relative proportion of sexual selection scores across each category of data certainty. Bars are coloured according to sexual selection score (SS) (blue = 0; red = 4). The data underlying this figure can be found at https://doi.org/10.6084/m9.figshare.27255609. (TIF) [file pbio.3002856.s004.tif]

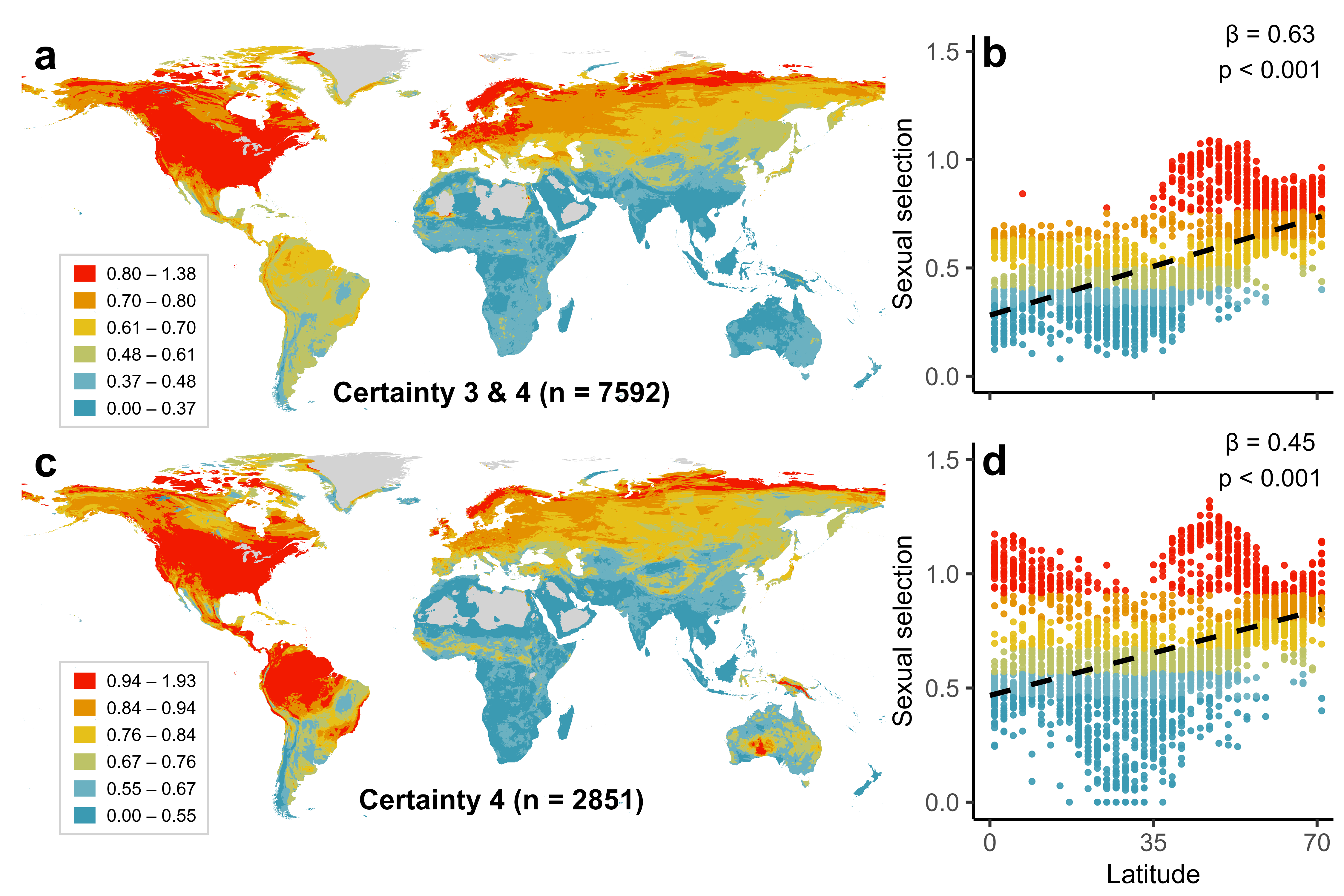

Supplement: S3 Fig — Upper panels show average sexual selection for a subset of species with higher-quality data (scored 3–4 for data certainty; n = 7,592) mapped globally (a) and plotted against latitude (b). Lower panels show average sexual selection for a smaller sample of species (n = 2,851) in the top category for data certainty (scored 4), again mapped globally (c) and plotted against latitude (d). In a and c, averages for each cell are calculated from all species with breeding range maps overlapping each 5-km grid cell. To aid visualisation, maps were coloured using discrete intervals with an equal number of cells. In b and d, points represent mean sexual selection per 200-km grid cell; dashed lines were generated from spatial simultaneous autoregression (SAR) models predicting mean sexual selection strength. To reduce noise, cells with <10 species were excluded from all plots and models. Results are plotted using geographical range polygons provided by BirdLife International (www.datazone.birdlife.org) cropped to Earth’s land-surface using the BIO1 climate layer (www.chelsa-climate.org). The data underlying this figure can be found at https://doi.org/10.6084/m9.figshare.27255609. (TIF) [file pbio.3002856.s005.tif]

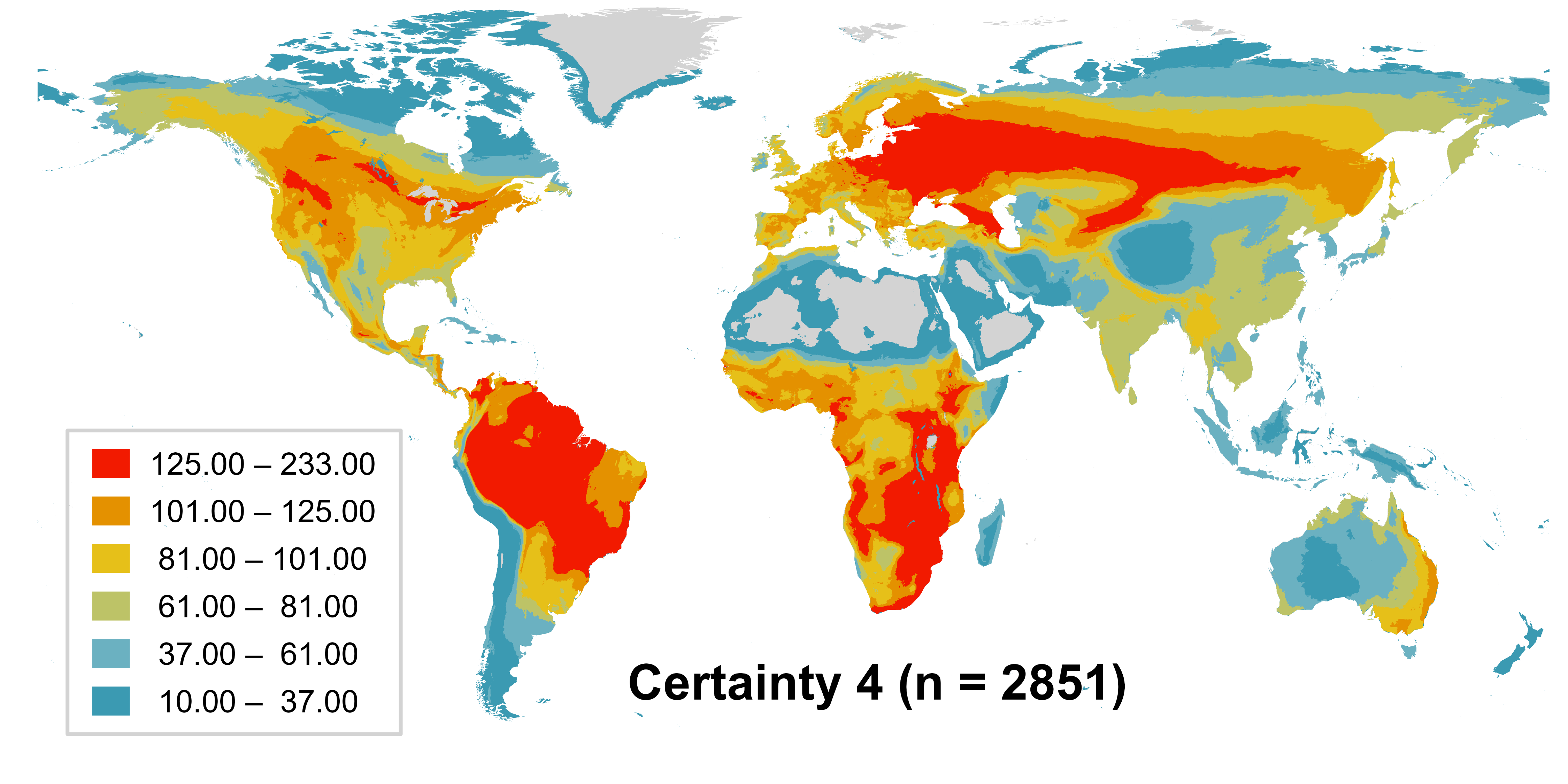

Supplement: S4 Fig — Worldwide species richness for 2,851 bird species with high data certainty (score = 4) included in a global phylogeny (www.birdtree.org [50]). Species were scored as 4 based on direct evidence published in primary and secondary literature (see Table A in S2 Text). Cell values represent the total number of high-certainty species, calculated by counting the number of breeding range maps overlapping more than 50% of each 5-km grid cell. To aid visualisation, cell values were grouped into equal-sized bins to reduce a skew in the colour scale caused by outlier cells with high species richness. Cells with <10 species were excluded. Results are plotted using geographical range polygons provided by BirdLife International (www.datazone.birdlife.org) cropped to Earth’s land-surface using the BIO1 climate layer (www.chelsa-climate.org). The data underlying this figure can be found at https://doi.org/10.6084/m9.figshare.27255609. (TIF) [file pbio.3002856.s006.tif]

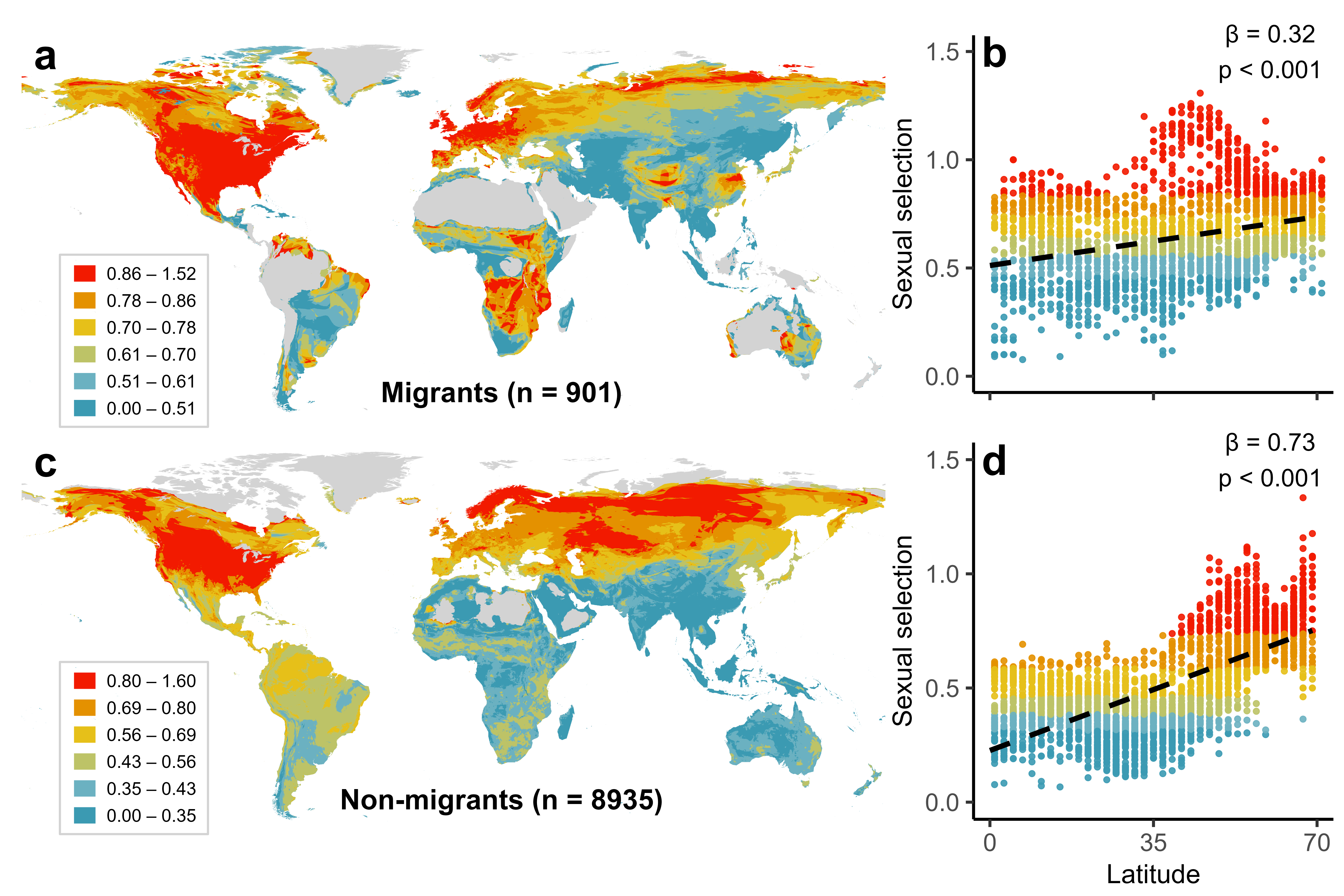

Supplement: S5 Fig — Based on occurrence data from the breeding range, upper panels show strength of sexual selection in long-distance migrants mapped globally (a) and plotted against latitude (b). Lower panels show strength of sexual selection in short-distance and resident species mapped globally (c) and plotted against latitude (d). Sexual selection was scored in 5 categories ranging from monogamy (0) to extreme polygamy (4; see Methods). In maps (a, c), averages for each cell are calculated from all species with breeding ranges overlapping each 5-km grid cell. To aid visualisation, maps were coloured using discrete intervals with an equal number of cells. In scatterplots (b, d), points represent mean sexual selection per 200-km grid cell; dashed lines were generated from spatial simultaneous autoregression (SAR) models predicting mean sexual selection strength. Additional SAR models on a conservative data set (certainty scored 3–4) showed similar patterns (see Table C in S2 Text). To reduce noise, cells with <10 species were excluded from all plots and models. Results are plotted using geographical range polygons provided by BirdLife International (www.datazone.birdlife.org) cropped to Earth’s land-surface using the BIO1 climate layer (www.chelsa-climate.org). The data underlying this figure can be found at https://doi.org/10.6084/m9.figshare.27255609. (TIF) [file pbio.3002856.s007.tif]

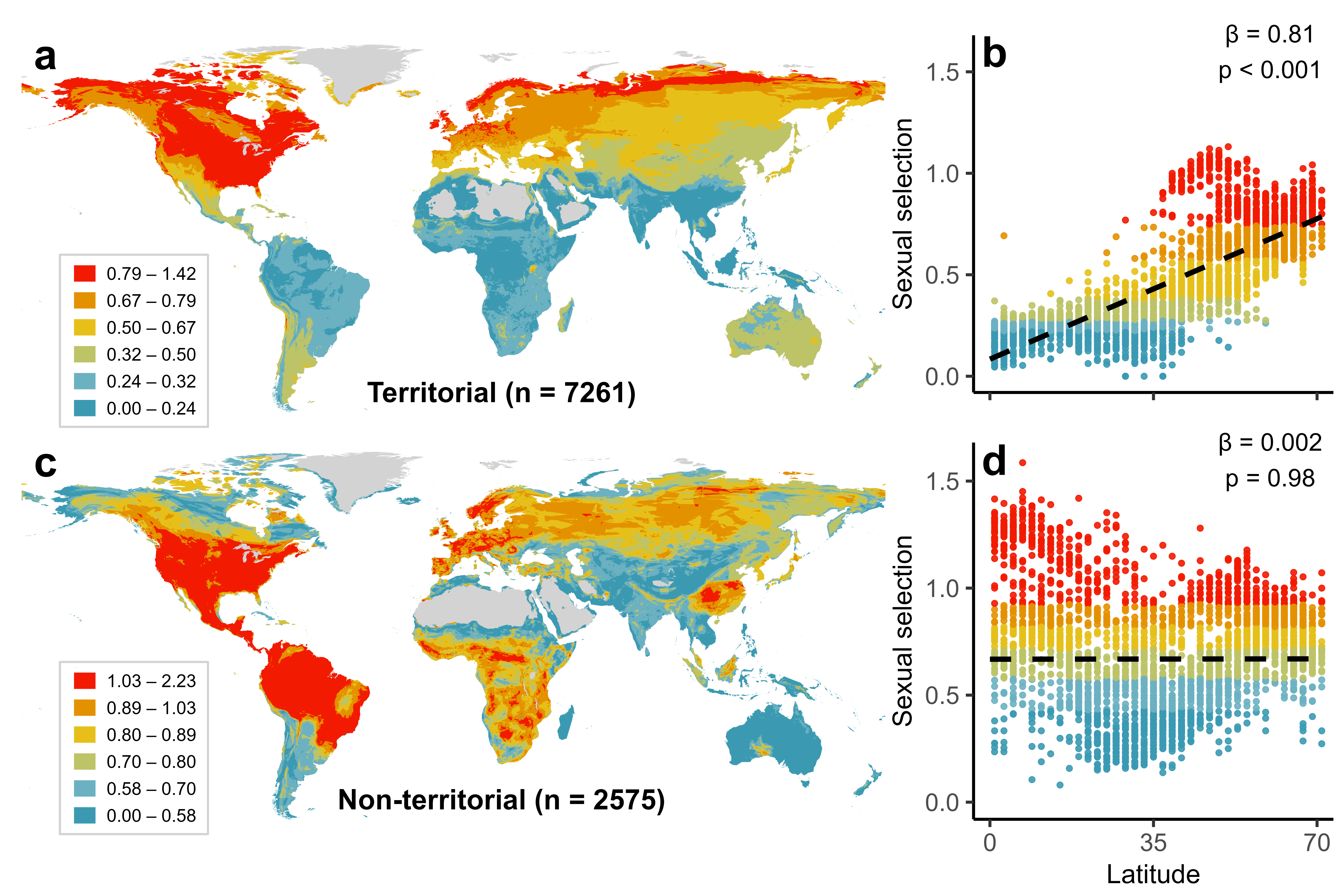

Supplement: S6 Fig — Upper panels show strength of sexual selection in seasonal and year-round territorial species mapped globally (a) and plotted against latitude (b). Lower panels show strength of sexual selection in non-territorial species mapped globally (c) and plotted against latitude (d). Sexual selection was scored in 5 categories ranging from monogamy (0) to extreme polygamy (4; see Methods). In maps (a, c), averages for each cell are calculated from all species with breeding range maps overlapping each 5-km grid cell. To aid visualisation, maps were coloured using discrete intervals with an equal number of cells. In scatterplots (b, d), points represent mean sexual selection per 200-km grid cell; dashed lines were generated from spatial simultaneous autoregression (SAR) models predicting mean sexual selection strength (see Methods). Additional SAR models on a conservative data set (certainty scored 3–4) showed similar patterns and are reported in Table C in S2 Text. To reduce noise, cells with <10 species were excluded from all plots and models. Results are plotted using geographical range polygons provided by BirdLife International (www.datazone.birdlife.org) cropped to Earth’s land-surface using the BIO1 climate layer (www.chelsa-climate.org). The data underlying this figure can be found at https://doi.org/10.6084/m9.figshare.27255609. (TIF) [file pbio.3002856.s008.tif]

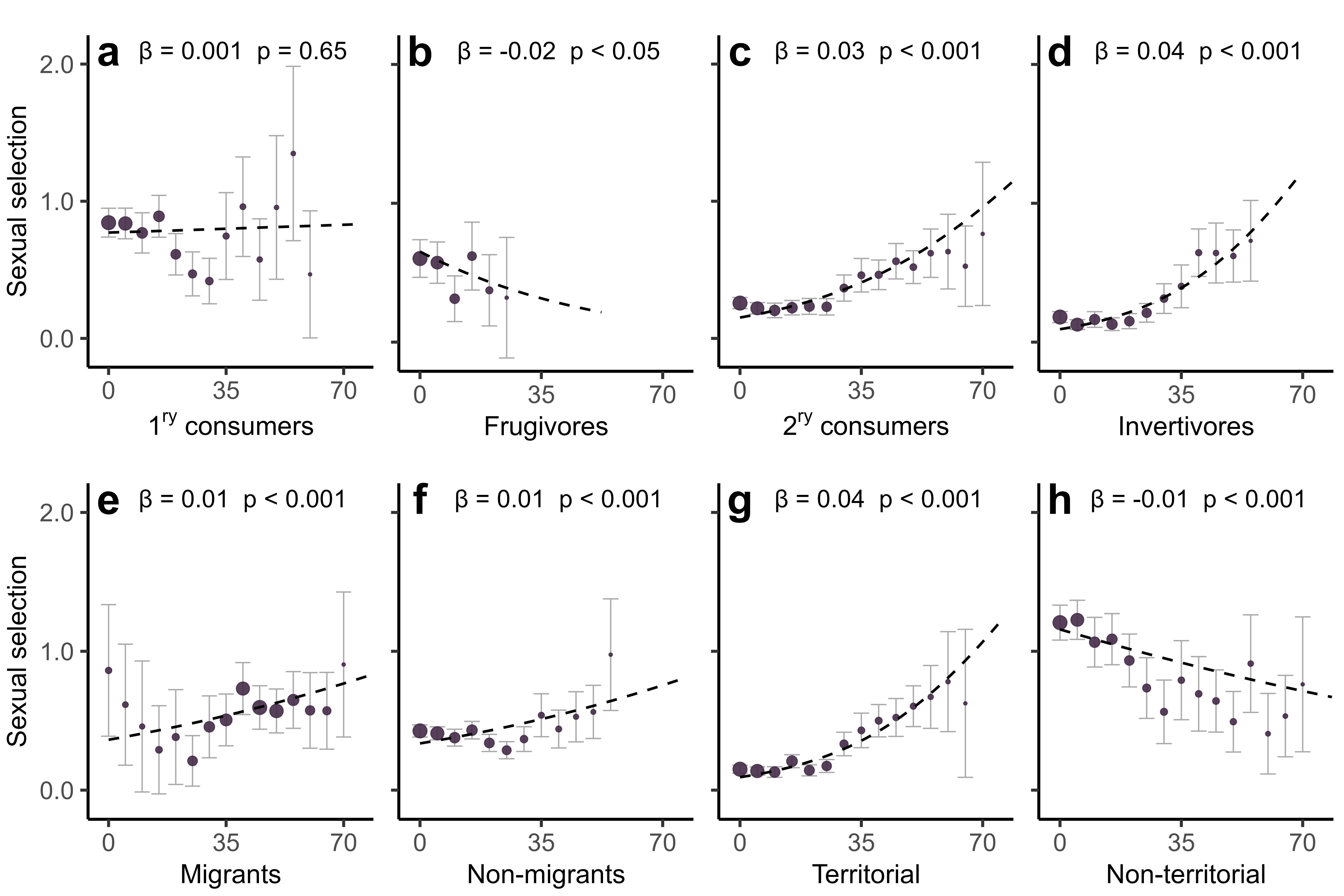

Supplement: S7 Fig — Panels show results of Bayesian species-level regression models predicting variation in the strength of sexual selection across latitude, with species samples partitioned by ecological traits. To aid visualisation, species were pooled into 5-degree latitude bins, based on the centroid latitude of their breeding ranges. Points denote mean sexual selection, scaled by the relative sample size of each 5-degree bin; bars denote 95% credible intervals; dashed lines were generated from species-level regression models (see Methods). To reduce noise, latitudinal bins with <10 species were excluded from all plots; model predictions extend across all bins containing species data. Additional species-level models restricted to a subset of species with higher-quality data (scored 3–4 for data certainty) showed similar patterns (Table D in S2 Text). The data underlying this figure can be found at https://doi.org/10.6084/m9.figshare.27255609. (TIF) [file pbio.3002856.s009.tif]

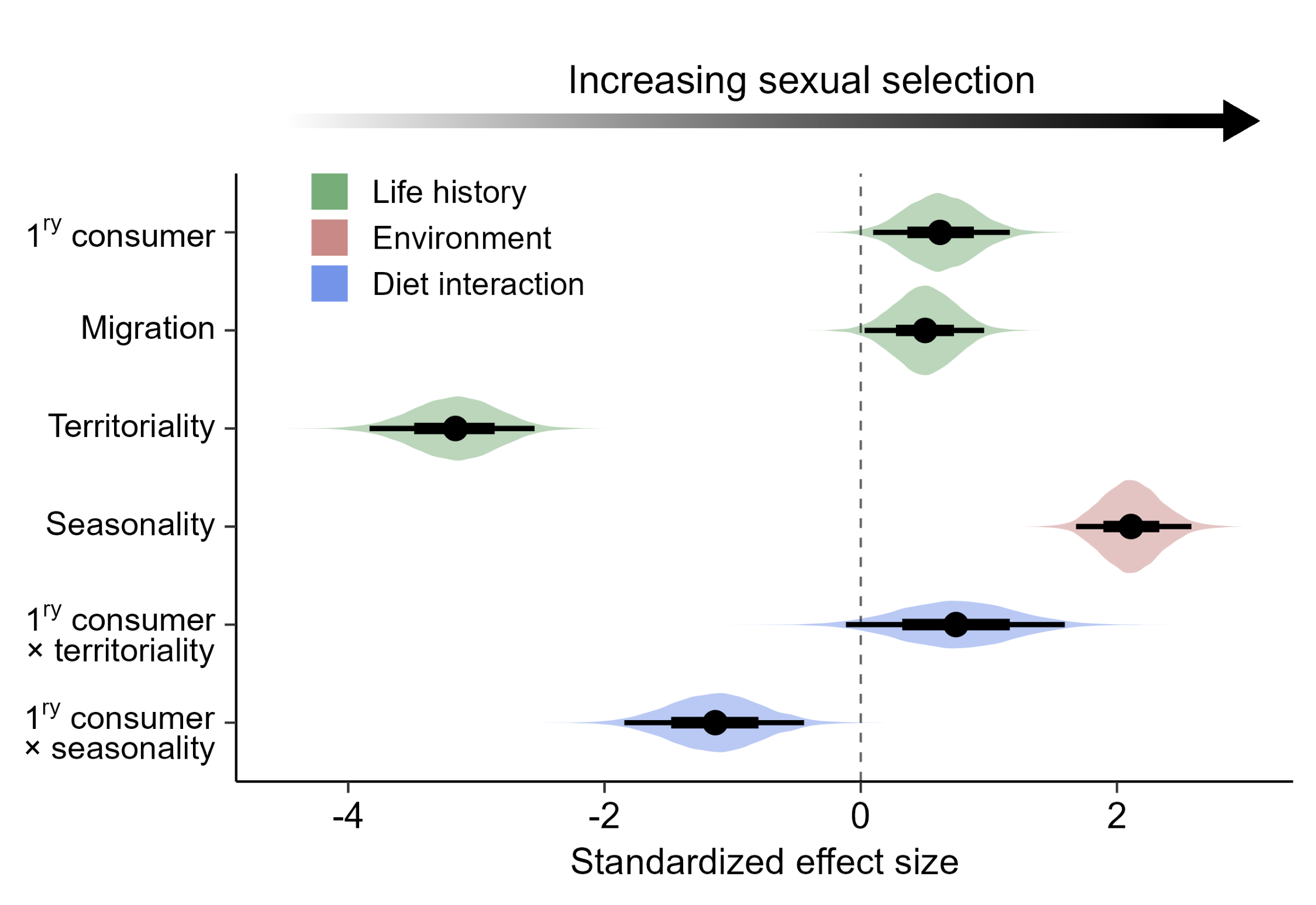

Supplement: S8 Fig — Results shown are from Bayesian phylogenetic models testing drivers of sexual selection in species scored with high data certainty scores (scored 3–4; n = 7,592 species). Predictors include 3 life history variables (green), 1 climatic variable (pink), and 2 key interactions between diet and the dominant effects (territoriality and seasonality; blue). The reference groups for the 3 categorical predictors are as follows: secondary consumer; no migration; and no territoriality, respectively (see Methods for definitions). Models were run on a sample of 50 phylogenetic trees extracted from www.birdtree.org [50], grafted to the Prum and colleagues [61] genomic backbone. Dots show mean effect size estimates from 12,500 posterior draws. For each effect, broad bases of whiskers show 66% credible intervals (CI); narrow tips of whiskers show 95% CI. Coloured distributions indicate the spread of effect size estimates, generated from a sample of 1,000 posterior draws. Full statistical results are presented in Table G in S2 Text. The data underlying this figure can be found at https://doi.org/10.6084/m9.figshare.27255609. (TIF) [file pbio.3002856.s010.tif]

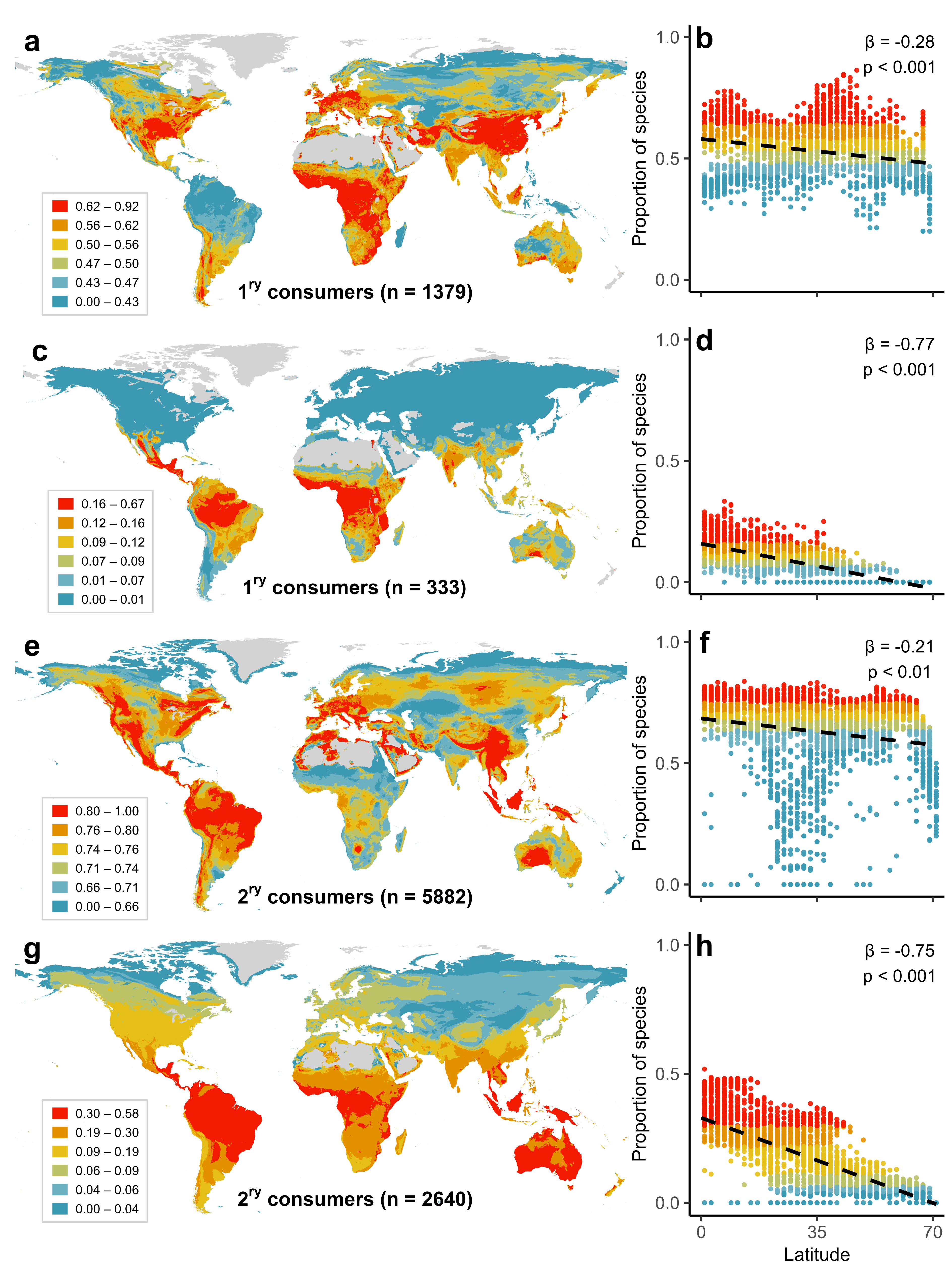

Supplement: S9 Fig — Proportion of territorial primary consumers mapped globally (a), and plotted against latitude (b), compared with those holding year-round territories only (c, d). Proportion of territorial secondary consumers mapped globally (e) and plotted against latitude (f), compared with those holding year-round territories only (g, h). Territoriality data (see Methods) were converted into binary scores for mapping purposes: in a, b, e, and f (0 = none; 1 = seasonal/year-round); in c, d, g, and h (0 = none/seasonal; 1 = year-round). In maps (a, c, e, g), averages for each 5-km grid cell are calculated from all species with breeding range maps overlapping each cell. To aid visualisation, maps were coloured using discrete intervals with an equal number of cells. In scatterplots (b, d, f, h), points represent mean sexual selection per 200-km grid cell; dashed lines were generated from spatial simultaneous autoregression (SAR) models predicting mean sexual selection strength. To reduce noise, cells with <10 species were excluded from all plots and models. Results are plotted using geographical range polygons provided by BirdLife International (www.datazone.birdlife.org) cropped to Earth’s land-surface using the BIO1 climate layer (www.chelsa-climate.org). The data underlying this figure can be found at https://doi.org/10.6084/m9.figshare.27255609. (TIF) [file pbio.3002856.s011.tif]
