# Supplementary material for: Climate and ecology predict latitudinal trends in sexual selection inferred from avian mating systems
Source: PLoS Biol. 2024 Nov 4;22(11):e3002856. doi: 10.1371/journal.pbio.3002856 (PMC11567637; doi:10.1371/journal.pbio.3002856)
Supplement: Attachment — Submitted filename: Barber et al. response_to_reviewers.pdf [file pbio.3002856.s016.pdf]

Reviewer #1:

It was a great pleasure to read the manuscript entitled "Climate and ecology predict latitudinal trends in sexual selection inferred from avian mating systems". The text is extremely well-written, and the overall theoretical question is clearly presented in the introduction. I carefully went through the methods and was able to follow all the analytical procedures performed by the authors. Despite the complexity of the analyses, the results are presented clearly, and the figures are especially effective in illustrating the general patterns. The discussion, although focused on birds, is interesting and places the obtained results in a broader context.

Given that I was not one of the previous reviewers, I carefully read all the comments and suggestions made regarding the first version of the manuscript. I agree with the authors' assessment that some of the criticisms seem to stem from a lack of familiarity with macroecological methods or biases against a macroecological approach in studies of sexual selection. While I do not identify myself as a macroecologist, I do appreciate the use of a predictive macroecological approach to understand how sexual selection may operate at large geographic scales.

The criticisms of using a proxy for sexual selection intensity made by reviewers 1 and 2 is valid, but the authors present compelling arguments in favor of the metric used. Additionally, they conduct various analyses to support the validity of this metric and test the robustness of the results in relation to data quality. However, the question remains: despite all the procedures adopted to address the reviewers' criticisms, do the results indeed provide useful information about macroecological gradients in sexual selection intensity? From my perspective, the patterns are solid and the study will undoubtedly not only stimulate discussions but also advance the field of knowledge. Therefore, I believe the work deserves to be published.

Next, I will offer some comments and suggestions on the manuscript. Although I am not an expert in birds and may not feel entirely comfortable evaluating the strength of certain taxon-specific arguments, I hope my input help the authors further improve the quality of the study. If you have any questions, please do not hesitate to contact me.

Glauco Machado
E-mail: glaucom@ib.usp.br

= = = = = = = = = = = = = = = = = = = =

MAJOR COMMENTS

1. The fact that diet modulates the strength of sexual selection in birds is one of the most interesting findings of the study. In the first paragraph of the discussion, the authors suggest that the observed differential response for primary and secondary consumers could explain discrepancies in the reported latitudinal patterns for insectivorous and frugivorous birds. Similarly, the authors propose that discrepancies in latitudinal patterns observed for other taxa could be explained by differences in diet. Unfortunately, I am not certain that the data obtained for birds can shed light on the other taxonomic groups mentioned in the cited articles. Saenz et al. (2006) worked with anurans, which are exclusively secondary consumers; Svensson & Waller (2013) worked with damselflies, also exclusively secondary consumers; Machado et al. (2016) worked with harvestmen, which are omnivores but with a strong tendency towards carnivory; and Krasnov et al. (2022) worked with fleas, which are blood-feeders. In other words, the four mentioned articles deal with taxa that should respond similarly to secondary consumer birds. However, both harvestmen and fleas respond similarly to frugivorous birds from my perspective. Therefore, variations in diet among taxa do not seem to be

the primary cause of differences in latitudinal patterns; the explanation may depend on other factors. I think the reproductive biology of birds presents two crucial differences in relation to the taxa mentioned above, hindering a direct comparison of the results: (a) scramble competition is a mating system absent in birds but extremely common in anurans, insects, and harvestmen; and (b) social and genetic monogamy is common in birds but extremely rare in anurans, insects, and harvestmen.

Response: We thank Prof Machado for his positive appraisal and thoughtful comments about our manuscript.

We agree that the link between diet and sexual selection is one of the more interesting results in our study, although diet is unlikely to be a direct driver of changes in sexual selection. Instead, dietary differences in birds often lead to differences in parental care (uniparental versus biparental care), with varying effects across latitudes. While this seems to explain differences in previous results and resolves much debate in the ornithological literature, the mechanisms may differ in some other animal groups, as noted by the reviewer. Nonetheless, we feel it is reasonable to suggest that our results point towards general explanations for variation in sexual selection in other animal groups, which are likely also driven by a complex array of interacting factors centred around climate and ecology. We have edited the text (lines 259-60) to clarify our point.

All line numbers were referenced using the clean version of our latest submission, without including track changes. A separate track changes file was also uploaded separately, which should align.

2) "While this may cause a positive link between territoriality and sexual selection in some avian systems, we find that the overall global pattern is reversed, presumably because resource defence polygyny is relatively rare." (L291-293) - OK, but please note that resource defence polygyny is relatively common in damselflies, a taxon in which the latitudinal gradient in the strength of sexual selection is positive (Svensson & Waller 2013), just like birds.

Response: We think our results reflect the abundance of stable monogamy in year-round territorial birds, a behavioural system which is abundant in tropical birds, and much rarer at high latitudes. We make no claims about generalisability to other animal systems. We have edited this sentence to clarify our meaning (lines 295-8).

MINOR COMMENTS

L11-12: the expression "mating systems" is already in the title. You could avoid redundancy changing this expression for "migration" or "temperature seasonality".

Response: Thank you for pointing out the redundancy in key words. We have replaced “mating systems” with “seasonality”.

L61-62: "...either resource defence polygyny or female-only parental care (Machado et al. 2016), leading..."

Response: We have now moved the citation to the end of the clause.

L72-73: "...as indices of strength of sexual selection..."

Response: We understand what the reviewer is driving at here. However, we find the suggested sentence structure a bit clumsy and we think that keeping the focus on sexual selection in general terms is valid here. Many of the papers we have cited explicitly used the metrics as indices of sexual selection, so we are simply reporting how the metrics were used in previous papers.

L85: "...inferred from mating systems across..."

Response: The reviewer may have a point that "mating behaviours" could be ambiguous or misinterpreted. We have edited the sentence to include their suggestion ("mating systems") but we also retain a reference to behaviour. This is because EPC and some other factors are not typically considered "systems" and also because we need to maintain the narrative transition from the previous paragraph, which explicitly sets up the need for behavioural observations.

L126: "...polygamy and EPP (Table 1; see also Methods)."

Response: We have now cited Table 1, which contains thresholds for polygamy and EPP.

L139-140: "...Clements et al. (2021) and BirdTree taxomomy..."

Response: In this instance, Clements refers to the name of the taxonomy, rather than the author, so we have not included the citation as suggested. To avoid confusion, we have switched the name of the Clements taxonomy to "eBird taxonomy", in line with the AVONET dataset (Tobias et al. 2022). We have edited the text to explain that eBird taxonomy is based on Clements et al. (2021).

L142: "...metrics of sexual selection commonly employed in the literature: residuals..."

Response: We have edited the sentence using the term "widely used" (line 143).

L150-151: "which provides a more accurate index of sexual selection (see Discussion)." - I suggest removing this part of the sentence.

Response: We agree that opportunity for sexual selection (*I*s) may not reflect sexual selection as a whole, but there is little doubt that it is a more accurate index of *pre-copulatory* sexual selection. Rather than removing this wording entirely, we have added a clarification that we are referring to pre-copulatory sexual selection.

L187: "...(negative) in frugivores (Fig. 4c,d)."

Response: We agree the direction needs clarifying. We have now included the suggested addition "(negative)" earlier in the sentence, where it seems to work better.

L191-192: "...in fruit-eating species (frugivores)..."

Response: OK – we have followed the reviewer's advice and removed "berry-eating"

L194: Both in the title and on lines 182/202/211/215/217-218/221/241 you refer to "ecology" or "ecological traits" (not life history). Please be consistent throughout the manuscript.

Response: We have now changed life history to ecological to be consistent with the rest of the manuscript.

L200-201: "...observed in lower latitudes (Fig. S6)."

Response: We have now changed "tropical regions" to "lower latitudes", to reflect a continuous decline in SS intensity across a wide range of latitudes.

L215-216: "(Tables S5-S6)"

L220: "(Fig. 5e; Tables S5-S6)"

Response: Done.

L249: "...that the strength of sexual selection..."

Response: Done.

L252: "...primary consumers (Fig. 4)."

Response: Done.

L266: "...birds was temperatue seasonality..."

Response: We have now changed "climatic" seasonality to "temperature" seasonality to be more explicit which aspect of climate we are referring to.

L326: I think this section would be better placed after the section "Sexual selection scores for all birds: validity, robustness and research priorities" and before the "Conclusions".

Response: We can see why the reviewer suggested this change but we have decided to retain the original placement of the section titled "Sexual selection in the context of climate change". We believe it follows logically from the previous section, about migration, which is clearly strongly linked to climate and temperature seasonality.

L360: why "sophisticated"?

Response: Rather than get bogged down in a debate about which metrics are more or less sophisticated, we have re-written this paragraph (lines 358 - 68) to explain that testes mass is not expected to reflect gradients of sexual selection whereas the other two widely used indices provide a more complete representation of the likely strength of sexual selection, even in lekking species.

L423: "...increases from 0 to 4 (Table 1)."

Response: We have now expanded this sentence, so it clear the defined scored clearly relate to the previous sentence (citing Table 1). See lines 428-431.

L467: "...mass (Pitcher et al. 2005..."

Response: Done

L566 and 902: et al. – italics

Response: Done.

Fig. 2: What do the colors represent in the graphics?

Response: The colour of points refers to increasing sexual selection. We think a key is unnecessary because the colours are simply replicating the y-axis, rather than providing new information. To avoid confusion, we have added an explanatory sentence to the caption.

Figs. 4b, 4d, 4h: I suggest limiting the dashed line to the segment of the x-axis for which there is available data.

Response: We have replaced this figure with SAR models which do not have the same problem of appearing to fit a model beyond the availability of data.

We still retain the figures referred to by the reviewer, but they have been redesigned and moved to the online material (Fig S7). The dashed lines continue to extend beyond the data points shown in some panels, so we have added text to the caption to explain that the model is fitted across all data, including hidden outliers.

L1036: "Clements et al. (2021)"

Response: Done.

L1069: "...Under Clements taxonomy, P. colchicus..."

Response: For clarity, we have changed our terminology to "eBird taxonomy" – which makes sense because the paper we cite is called "The eBird/Clements checklist of birds of the world".

L1130-1141: In cases where the Is was separately calculated for males and females of a given species, how was this information used in the database?

Response: When Is measures were available for both sexes, we used the highest Is value, regardless of sex. This makes sense as it reflects the maximum sexual selection experienced by individuals of the species. We used the same procedure when Bateman gradients were also calculated for both sexes. We now explain our procedure clearly on lines L1174-1177 in the section titled "Sexual selection metrics".

L1276: "...diverse species. Second..."

Response: Done.

Figs. S5d, S9b, S9d: I suggest limiting the dashed line to the segment of the x-axis for which there is available data.

Response: Dashed lines refer to species level models and therefore extend to the maximum latitude for all species. We now explain this in the relevant figure caption (fig. S7).

Reviewer #2:

** Please note that I have not read the reviews and authors replies for the previous submission of this manuscript to a different journal, that authors have attached with the present manuscript. As I see it, my job as a reviewer is to assess the manuscript submitted to PLoS Biol as it stands, and the manuscript's history of previous reviews and revisions is not relevant. The present ms with its supporting material should stand on its own, and I believe that this alone is what I need to assess.

The ms describes global patterns of geographic variation in the prevalence of sexual selection in birds. This has been done (or attempted) before several times, and in the introduction the authors make a strong case for why this is interesting and important for our understanding of evolution. They do an excellent job of clearly laying out the various alternative hypotheses to explain the possible patterns you could see. This study is probably the most thorough investigation of this issue to date, because it is based on comprehensive global datasets of bird geographic distributions and phylogeny, and the authors have constructed a novel dataset (from secondary data sources) that classifies all bird species into an ordinal-scale classification of sexual selection. They explore the geographic patterns for all birds species and for a number of ecological subsets of birds that allow them to explore nuances in the general pattern, and offer supporting evidence for or against the various mechanistic hypotheses. All in all it is an interesting and rigorous piece of work.

Response: We are grateful for the positive feedback and we also thank the reviewer for their more robust critique below as this has led to some major improvements to the figures and statistical analyses.

That said, I have a few worries about the degree to which the results support the conclusions the authors draw, which I will list below.

1. Much of the interpretation of latitudinal gradients in SS seems to be based on the p-values of the regression models. This is not ideal as in many cases the huge sample sizes are generating small p-values even when effect sizes are small and the visual pattern (from the maps and the plots) makes a latitudinal trend seem dubious.

Response: We understand the reviewer's scepticism about p-values generated from massive sample sizes. This is a widely held view, particularly among researchers that typically work with smaller samples, or experimental datasets. However, small effect sizes and low r-squared values are typical of large comparative analyses since macro-scale

patterns are inherently noisy (Møller & Jennions, 2002). Significant results still reflect a gradient or effect.

In the new draft of this manuscript, the analyses referred to by reviewer #2 have been moved online because we followed their useful suggestion to improve our statistical analysis. Specifically, to account for autocorrelation and to avoid problems with phylogenetic non-independence, we have replaced the previous species-level models with Spatial simultaneous auto-regression (SAR) models (see below). One advantage of the new modelling framework is that we now properly scale response variables, so that effect sizes are now shown on a typical 1SD scale. The new results show much more convincing gradients of sexual selection.

For example, Figure S3D certainly doesn't look like a latitudinal trend to me, even though the p-value is <0.05 and the authors are happy to take this as support for a positive association holding up for the species with the highest data certainty (line 168-173). I think they just need to be a bit more circumspect with their claims of support for latitudinal trends - use the visuals and effect sizes, not just the p-values, to draw these conclusions.

We agree that the previous version of figure S3D made a latitudinal gradient seem dubious. This was in part because we summarised data using means and error bars, without showing the underlying spread. We have now updated figure S3D to show the results of our new SAR models on the highest quality data, including all the underlying data points rather than averages/error bars. It is now much easier to see the latitudinal gradient and how this matches the updated SAR model predictions.

Overall, we agree with the reviewer that we needed to place less emphasis on p-values, and we hope these new figures and stronger effect sizes help to reinforce our main points.

2. Even with the visual patterns the authors need to be more cautious in their interpretation, or their acceptance of what looks like a trend. For example, in Figure 3C there is a prominent "hotspot" of SS in the Nullarbor Plain / Great Victoria Desert region of south-central Australia, which is a bit odd to say the least, because I'm fairly sure there are few endemic species here, most species that overlap this area are widespread across the southern arid zone, and there's no reason to think the assemblage here should have higher SS on average than surrounding regions. What I think is going on here is that this is an extremely poorly sampled region and this hotspot is likely driven by just one or two species whose ranges overlap the area and which happen to meet the higher data quality and SS criteria. More generally, this makes me wonder how "volatile" these geographic patterns are, ie to what degree the patterns in many parts of the world are driven by small numbers of species. It would be reassuring to know the richness of grid cells for each of these maps. This also makes me question the reliability of some of the analyses based on the aggregate geographic data.

Response: The reviewer is right that the Nullarbor Plain/Great Victoria Desert region pops out in figure S3C because it has low sampling of high-certainty species, and the apparent high SS is driven by some well-known species occurring in the area, such the emu. We now include a figure to show the species richness of high-certainty species which shows the regions where sampling is poorest (Figure S4).

On the other hand, the concerns raised about "reliability" of our models is unfounded. The reviewer seems to assume that our analyses are based on grid-cell data whereas the only grid-cell analysis is the SAR models that we have now included based on comments by the same reviewer.

The maps we include are meant to aid visualization and we use several different analytical approaches to ensure that our results are robust. While there are always going to be outliers, such cases are not really a problem for our analyses (or indeed any macroecological analysis) as they do not explain the overall patterns. Moreover, most of our analyses are at the species level, using centroid of species ranges to define our latitudinal gradient. These models are not sensitive to the types of biases that the reviewer describes. Thus, all results based on analyses using "aggregate geographic data" are supported by additional species-level models, as well as sensitivity analyses on high-certainty data subsets.

3. This leads on to an even broader concern. I wonder to what degree the global patterns of SS really are driven by latitude (well, the things that correlate with latitude, obviously), or are simply the results that emerge from the history of radiation of various large bird clades with differing degrees of SS, in different parts of the world - in other words, a pattern driven more by historical contingency than by mechanistic associations with the correlates of latitude. The phylogenetic conservatism of SS (lines 208-209) would seem to support this. I seem to recall this was also the conclusion arrived at by Jetz et al 2012 with respect to global variation in diversification rates. I know the authors have gone some way to addressing this through the inclusion of phylogenetic covariances in their models, but one concern I have is that the models are not spatially explicit (and I don't really see how dichotomizing latitude removes spatial autocorrelation, as suggested in lines 556-558). Phylogenetic and spatial effects are themselves strongly intercorrelated, and really, the only way to handle this in a regression-type model to extract any true underlying geographic pattern is to use methods that incorporate both phylogenetic and spatial covariances. Two examples of how this can be done are (1) Hua et al (2019) Nature Communications 10 (1), 1-10, and (2) Dinnage et al (2020), Proceedings B

287 (1926). The latter paper also addresses another problem the authors mention, which is how to handle species-level and geographic variables in the same model.

Response: This was a very useful critique which has led to a large amount of work re-analysing and re-mapping our data. We broadly agree the dichotomisation between tropical and temperate species did not effectively remove spatial autocorrelation. We also agree that our initial global analyses had not accounted for spatial autocorrelation in any other way, which limited our ability to dismiss historical contingency as the primary driver of modern patterns of sexual selection.

The reviewer suggests that we should tackle this issue using methods incorporating both phylogenetic and spatial covariances within the same regression-based model. We think that is a nice idea but unfeasible given the size of our dataset. The two exemplar references supplied by the reviewer had a much smaller and more accommodating sample size (n = 366 species in Hua *et al.*; n = 152 species in Dinnage *et al.*). Asking us to scale this computationally intensive approach to a sample of 10,000 species is not realistic, particularly as our response variable is an ordinal ranking (unlike the continuous variables in the Hua and Dinnage studies). Instead of a combined model, which could take years of parallel processing power to converge using suitable phylogenetic methods (Nakagawa & De Villemereuil 2019), we now account for spatial and phylogenetic auto-correlation separately, following methods adopted in numerous global analyses (e.g. Cooney *et al.* 2022; Rabosky *et al.* 2018).

To address spatial concerns, we (1) removed the tropical versus temperate analyses entirely on the grounds that they don't add anything substantive, and (2) updated our main analyses of latitudinal gradients (species-level regression models) with spatial simultaneous auto-regression (SAR) models to account for spatial autocorrelation. For our modelling framework, we followed the methods used by Cooney et al. (2022), who faced similar challenges addressing spatial autocorrelation in global gradients.

In these new SAR analyses, we used average sexual selection per 200kmsq grid cell as the response variable, and distance from the equator as the predictor. 200kmsq was chosen as a compromise between scale and computing power; we feel the scale works well in the new scatterplots, showing much more convincing latitudinal gradients in our dataset, and more clearly reflecting our statistical results. The new results are highly consistent with the previous species-level models, with the advantage that they now also show the underlying distribution of sexual selection across grid cells. We have updated the main methods (L548-75) and supplementary methods (L1219-42), as well as figures 3-4, and supplementary figures S3, S5-7 and S9.

Rather than completely remove the previous species-level Bayesian latitudinal models, we now include them in the supplementary material. We think they are useful in showing species-level analyses which support the grid-cell analyses, generating similar gradients.

The other main concern of Reviewer #2 was that our results could be explained by the "history of radiation" of bird clades. To directly address this question, we ran a series of univariate and multivariate phylogenetic analyses (shown in figure 4 and 5, and presented

in detail in both the results and discussion). By explicitly including phylogenetic information from the global bird tree (Jetz et al. 2012) into our models, our hypothesis-testing procedure controls for the effects of historical contingency.

4. Line 148-151: I applaud the authors use of these three alternative SS metrics to do a validity check on their index, but generalizing from 14 species to all 10,000 bird species seems to be pushing inductive inference to (beyond?) its limits.

Response: On one hand, we accept the reviewer's point that a sample of 14 is not exactly ideal. We searched intensively for relevant estimates of Bateman gradients, but they are rare in the literature. On the other hand, the sample contains contrasting cases and they line up pretty well with our scoring system. Also, n = 14 is enough to run a valid statistical model. Ultimately, estimates of Bateman gradients are perhaps the most robust and relevant metric, so we think they are worth including as additional support. Rather than ditching the strongest empirical comparison, we prefer to retain all three correlations, particularly as the increasing tightness of the correlations makes sense – ie a weaker correlation for testes size because of the expected reduced testis in lekking species, and a closer estimate for metrics of mating variance (Bateman gradients and OSS). Fortunately, the other tests have better samples (n = 100 species for OSS; n = 1000 species for residual testes mass).

5. Figure 2: what do the three colours represent?

Response: The colours in this graph represent the five sexual selection scores (0-4) for the species in our dataset. We chose not to include an additional colour legend, as the colours are simply repeating the y-axis. Nonetheless, this issue was also noted by reviewer #1, so to avoid confusion we have added an explanatory sentence to the figure caption.

6. Figure 4C: can you really interpret the frugivore pattern as a negative latitudinal gradient, when the data are virtually confined to the tropics?

Response: Although the frugivore gradient is truncated, it is still a gradient. The sample includes >1000 species and the tropics is a very broad region. Moreover, avian tropical species tend to have smaller ranges compared to temperate species, so it's possible to identify latitudinal gradients at smaller scales. We think the new maps and SAR model data are now more convincing in this respect.

7. Conclusion: this seems a bit weak, without a clear take-home message, as if it was tacked on at the last minute. I can't offer any specific suggestions but I'd encourage the authors to think a bit more deeply about the general conclusions and broader significance of their findings. I would also remove the bit about relevance to climate change and the future of biodiversity, which seems a bit confected (and unnecessary).

Response: We feel that short and snappy conclusions are sometimes a strength rather than a weakness. However, we can see that the previous version of the conclusions may have seemed to gloss over some issues and over-emphasise others. We would rather not

over-elaborate this section, so instead we have focused on making the key points in simple language. We state that we have shown how multiple interacting traits can drive spatial gradients in sexual selection, sometimes with opposite expectations in different types of species. We believe this is a fairly important contribution which helps to explain why previous authors identify contrasting latitudinal gradients in sexual selection. We also make clear that, along with the underlying data, the findings provide a framework for testing a wide array of hypotheses.

Regarding the apparent tacking-on of the mention of climate change, we think the reviewer makes a reasonable criticism, so we have edited the last sentence of the main text to expand our point and clarify the relevance of our study.

References

Cooney, C.R., He, Y., Varley, Z.K., Nouri, L.O., Moody, C.J.A., Jardine, M.D., et al. (2022). Latitudinal gradients in avian colourfulness. *Nat. Ecol. Evol.*, 1–8.

Dinnage, R., Skeels, A. & Cardillo, M. (2020). Spatiophylogenetic modelling of extinction risk reveals evolutionary distinctiveness and brief flowering period as threats in a hotspot plant genus. *Proc. R. Soc. B Biol. Sci.*, 287, 20192817.

Hua, X., Greenhill, S.J., Cardillo, M., Schneemann, H. & Bromham, L. (2019). The ecological drivers of variation in global language diversity. *Nat. Commun.*, 10, 2047.

Møller, A. & Jennions, M.D. (2002). How much variance can be explained by ecologists and evolutionary biologists? *Oecologia*, 132, 492–500.

Nakagawa, S. & De Villemereuil, P. (2019). A general method for simultaneously accounting for phylogenetic and species sampling uncertainty via Rubin's Rules in comparative analysis. *Syst. Biol.*, 68, 632–641.

Rabosky, D.L., Chang, J., Title, P.O., Cowman, P.F., Sallan, L., Friedman, M., et al. (2018). An inverse latitudinal gradient in speciation rate for marine fishes. *Nature*, 559, 392–395.
