# Supplementary material for: Climate and ecology predict latitudinal trends in sexual selection inferred from avian mating systems
Source: PLoS Biol. 2024 Nov 4;22(11):e3002856. doi: 10.1371/journal.pbio.3002856 (PMC11567637; doi:10.1371/journal.pbio.3002856)
Supplement: Attachment — Submitted filename: Barber_et_al_response to reviewers.docx [file pbio.3002856.s014.docx]

**Decision, Review Comments and Author Responses for NCOMMS-23-41241-T**

The following sections show the editorial decision letter and reviewer comments sent by *Nature Communications*. We have briefly outlined our responses, highlighting where the previous reviewers have misunderstood the research and where their comments have led to corrections, clarifications and other improvements to the revised ms.

Some of the misunderstandings were caused by the reviewers being unfamiliar with macroecological studies and their core assumptions. It would be helpful if any further submission was reviewed by at least one macroecologist.

**Editor's Comments to Author**

Dear Mr Barber,

Your manuscript entitled "Resolving the climatic and ecological drivers of geographical gradients in avian sexual selection" has now been seen by 3 referees, whose comments are appended below. Considering their advice I regret to inform you that we cannot publish your manuscript in Nature Communications.

You will see that, while the reviewers find your work of interest, they raise substantive concerns that cast doubt on the advance your findings represent over earlier work and the strength of the novel conclusions that can be drawn at this stage. Unfortunately, these reservations are sufficiently important to preclude publication of this study in Nature Communications.

I know that this decision will disappoint you, especially in view of several positive comments. I should explain, however, that we do not ask referees to say whether they think that a paper belongs in Nature Communications. Instead, we ask them to tell us specifically what they feel to be the paper's contribution, and what its significance might be, and based on these comments and our own editorial criteria, we decide whether publication in Nature Communications is appropriate.

Although we cannot publish your paper, it may be appropriate for another journal in the Nature Portfolio. If you wish to explore the journals and transfer your manuscript please use our manuscript transfer portal.

**Reviewer #1 (Remarks to the Author):**

The paper presents a global analysis of “the strength of sexual selection” in bird species and identifies latitudinal gradients in this variable for subsets of species. The paper also identifies some correlates of these gradients, which could be largely grouped into two sets of variables: those associated with seasonality and those associated with territoriality or resource defense. Although I see the value in the compilation of this global data set for all birds, I have some serious concerns about the authors’ approach of merging very different, and often independent, components of the reproductive systems of species into a one-dimensional, five-point score of increasing strengths of sexual selection.

First of all, what is sexual selection? In the opening sentence of the introduction, the authors mention the peacock’s tail and the song of the nightingale as examples of a “pervasive mechanism driven by competition among individuals for reproductive success (Andersson 1994)”. This is an ok definition as it puts sexual selection in the context of trait evolution, although many workers in this field would narrow down reproductive success to mating success or fertilization success to distinguish them from components of reproductive success driven by natural selection (see e.g. Shuker & Kvarnemo 2021 in Behavioral Ecology and multiple commentaries from leading experts). This discussion may seem somewhat semantic, but my point here is that sexual selection explains the evolutionary change in traits, and that the strength of sexual selection cannot be measured without reference to a trait. The raw material for sexual selection is of course variance among individuals in mating success (or fertilization success) and its covariance with a trait, but this variance is only a measure of the “opportunity for sexual selection”. There can be high variance in mating success and no sexual selection if there is no phenotypic trait associated with the mating variance. So, the strength of sexual selection can only be measured in its effect on a trait. Typical traits are size, displays, ornaments, or colors in birds, usually expressed in units of sexual dimorphism, assuming that sexual selection has stronger effects on the sex that competes more strongly for matings. The way I read the current paper is that it does not provide a measure of the “Strength of sexual selection”. Rather it attempts to classify species into categories of increasing “opportunity for sexual selection”, which is conceptually a very different variable.

**Response: We have changed the wording in the title and throughout the ms to highlight that we are not claiming to measure sexual selection directly, but an index of sexual selection based on avian mating systems. The use of mating systems in this way is widespread in the literature, so we provide additional rationale for our approach in the context of previous research (lines 84-93; 399-417). In addition, we have simplified the score so it just takes into account two well-established components of the mating system: polygamy and extra-pair paternity (the reviewer agree these are indicative of what they call “opportunity for sexual selection”).**

**One point we disagree with is the view that sexual selection can only be relevant in relation to a trait, and that morphological attributes like sexual dimorphism is a better way of measuring sexual selection. This has repeatedly shown to be false, or at least a weak general proxy of sexual selection in birds for a variety of reasons that we now explain in the introduction. Indeed, the inability of standard approaches like sexual dimorphism and sexual dichromatism to capture sexual selection in birds is one of the main motivating factors behind our dataset.**

**We have edited the text in several places to acknowledge the limitations of our metric and to point out that it is nonetheless the best available metric for a global synthesis of sexual selection. On Line 93, we explicitly state: “Clearly, more direct measures of sexual selection would be preferable, but such measures are currently only available for relatively few species, and therefore not suitable for exploring global gradients.”**

Second, the five scores of sexual selection, as defined in the Extended Data Table 1, combines three sets of variables: 1) the social breeding system, from monogamy through increasing frequency of polygamy to lek breeding, 2) increasing frequency of extrapair paternity, and 3) skew in the operational sex ratio. Again, these are variables that may carry a signal of the opportunity for sexual selection at various stages of the reproductive cycle (pair-bonding success, copulation success, paternity success), but they do not tell whether there is sexual selection on a particular trait. To the contrary, they probably signal an opportunity for sexual selection on quite different traits, which means that forcing them into a single ordinal scale may obscure any general patterns. For example, there is ample evidence in birds that male plumage or song traits can be important in mate attraction and pair formation, but there is no clear evidence that extrapair paternity is explained by the same traits. The only male trait that consistently explains paternity success in birds is male age and that has probably nothing to do with sexual selection at all, but should instead be considered a life-history trade-off between reproductive effort and survival shaped by natural selection. The age-related paternity also implies that the variance in male paternity measured among males of different ages within a single season, gives an inflated measure of the variance among males in life-time paternity success (selection will only work on life-time fitness). There are also many reports of reciprocal paternity among males (usually between neighbors) which in effect reduces variance among males in paternity success.

**Response: We agree that including operational sex ratio as a component of our score may have been confusing to some readers, so we simply removed this information from our scores to streamline the metric. Only a few species changed score and these changes did not alter the conclusions in any re-analysis.**

**We retained extra-pair paternity (EPP) because, contrary to the view of the reviewer, there is general agreement that EPP is a valid component of sexual selection linked to changes in mating variance across bird species. We cite papers validating this assumption.**

**Overall, the criticism of the reviewer seems to rest on their unfamiliarity with the macroecological method and literature. Our scoring system is a clear improvement of a well-established system for estimating the strength of sexual selection used for decades in macroecological analyses, as we now point out in the introduction (lines 86-88). Moreover, we believe that almost all readers will interpret the strong correlations between our scores and other quantitative proxies for sexual selection (Fig 2) as compelling evidence that we are at least capturing a major component of “sexual selection” as conceptualised by the majority of evolutionary biologists.**

**Unfortunately, there is no catch-all metric of sexual selection for all birds, and any given system will always be some species which do not fall neatly within pre-defined criteria. However, the argument that our score will “obscure any general patterns” is an overstatement and seems to be formulated by someone with little feel for macroecological methods. We argue the exact opposite that capturing more than one dimension of sexual selection in our score is an advantage precisely because it is not designed to disentangle specific mechanisms but to identify general patterns. We now make this point in the main text (lines 70-83).**

The current grading systems has an implicit assumption that the grading of the social mating system has a parallel increase in the frequency of extrapair paternity. This is far from true. There are several examples of socially monogamous species with extremely high levels of extrapair paternity. Likewise, there are lekking species where females never copulate with more than one male (no sperm competition). And there is a lot of variation between these extremes, which means that these are two independent or uncorrelated sources of variation in male fitness.

**Response: We are very surprised that the reviewer makes this point as this is a serious misunderstanding of how our scoring system works. We are, of course, well aware that sexual selection may map differently onto the different dimensions of our system, just as the reviewer describes. However, this is not a problem because there is no need to assume a correlation between the different elements as we simply take the highest score triggered by any criterion. In other words, we use the same approach as most macroecological scoring systems, including the IUCN Red List. For example, in the case mentioned by the reviewer, a species may form leks (score = 4) and have zero EPP (score = 0). In this case, we simply use the highest score (4). We had thought this was self-evident and we agree that it would be useful to spell this out clearly for readers less used to macroecological scoring systems. To make this point, we have edited text in lines 436-439 and in the caption to Table 1.**

Given these considerations, I am skeptical to the current approach of a global analysis of the strength of sexual selection by comparing “apples and oranges” as a fruit cocktail. It fogs up the problem instead of clarifying it. Much better to analyze the evolution and determinant of social mating systems and of paternity patterns as separate phenomena, as done by a number of previous studies.

**Response: We understand the concerns expressed by reviewer #1, but feel they are misrepresenting the purpose of our study. We have slightly simplified the scoring system in response to their comments (see above), but we still retain lines of evidence from social and genetic mating system in our proxy for sexual selection. Genetic information is becoming increasingly available for bird species, we think it would be inappropriate to ignore this source of information when generating our scores. Nonetheless only ~5% of bird species are included in genetic studies, so it would seem negligent to ignore extra-pair behaviour, which is reported for many more species. We believe that additional sources of evidence do not cloud the issue, but provide more texture to our scoring system, with the major advantage of not pooling all socially monogamous species as one category (which raises statistical problems is this is such a large subset of species). Overall, we believe our composite scores are the best available system for evaluating the potential for sexual selection across a global sample of species.**

**Reviewer #2 (Remarks to the Author):**

This paper contains an excellent analysis that nicely resolves the disparate previous claims that indices of the intensity of sexual selection varies positively, negatively, or not at all with latitude. You have correctly highlighted that result at the beginning of your Discussion and I recommend that you focus on that as the main contribution of this research. As did those other studies, you have used indices for the intensity of sexual selection that are, in my opinion, only marginally useful for interspecific comparisons, despite the weak relationships uncovered. Thus I would prefer to see you speculate on the relationships (correlates) with territoriality etc with those indices (as you have done), rather than making bold claims about their influence on the intensity of selection. The bottom line is that I think there are aspects of this work that are quite valuable but there is much about the presentation that I find wanting (see details below) and I remain unconvinced that your study helps us to understand the important drivers of sexual selection across the entire radiation of birds.

**DETAILS**

Title: This seems a bit over-hyped to me. As good as your study is, it is far from resolving these drivers. I feel the title would be more accurate if it omitted the first two words. I also wonder whether ‘drivers is appropriate here as you have really uncovered patterns but without some further work it is hard to know whether those correlates indicate anything causal.

**Response: Fair points. We have now changed the title to eliminate claims of causality and to explicitly state the assumptions of our study.**

16 by ‘its underlying drivers’ do you mean sexual selection’s drivers or the drivers of geographical variation.

**Response: We are referring to the underlying drivers of “geographical variation in this evolutionary mechanism” – the sentence is correct, and both interpretations mentioned by the reviewer are correct, so we have left the wording unchanged.**

25 explained by OR correlated with??

**Response: We think this is a semantic quibble so we kept the word “explained” in the abstract. Standard terminology in comparative analyses is to say that models “explain” the variance in the response variable.**

51 you seem to be using the term ‘sexual selection’ to mean ‘the intensity of sexual selection’. I suggest being more explicit as well as stating clearly what you mean by ‘intensity’

**Response: Agreed. We have since changed the text to be more explicit, which now says “The intensity of sexual selection”.**

61 presumably because both are correct, as you show in this paper

**Response: Yes. This is one of most important findings, with latitudinal gradients changing direction depending on the ecological guild within birds that we analysed. In that regard, our results are important because they resolve seemingly conflicting views and show that they stem from similar mechanisms.**

69 presumably, again, you are referring to intensity. Depending on the nature of constraints, dimorphism and trait elaboration might not be good measures for the intensity of selection. And, I would argue, the nature of those constraints can also influence EPP and polygamy independent of the intensity of selecton.

**Response: We agree that evolutionary constraints can equally influence the extent of sexual traits and sexual behaviours. We now acknowledge the limitations of mating systems in greater detail in the main text (lines 89-95). We would also argue that by publishing our dataset, we can allow researchers to choose the best proxy for sexual selection depending on the taxonomic clade and hypothesis in question.**

77 gradients?? latitudinal or selection/Bateman

**Response: We changed our wording to say “sexual selection gradients”.**

77-78 why?? Stating this does not make it true

**Response: We think the reviewer has misunderstood our point. Our statement is as follows:**

**“A more complete test of sexual selection gradients and their drivers requires more direct evidence from behavioural observations and parentage analyses, yet this information has not previously been available at the required taxonomic and geographic scale.”**

**The point we are making is perfectly accurate: that many macroecological studies use morphological/plumage features as indirect indices of sexual selection whereas more direct measures (based on mating systems and behaviours) are needed because of the large problems previously noted with the morphological approaches (which we cite extensively). Our study bumps up the sampling for these “more direct” metrics from a few 100 to over 10,000 species. Clearly this is a massive contribution and allows the first assessment of global pattern in a standardised measure of sexual selection.**

82 ‘strength of sexual selection’?? Is this the same as what you called ‘intensity’ above? How is ’strength’ defined?

**Response: We removed “strength” as to not introduce additional terms.**

96 but many studies of EPP provide no evidence that sexual selection is involved at all. In other words, there is no male trait associated with the incidence of EPP.

**Response: In our analysis comparing our scores against alternative metrics of sexual selection (Lines 147 – 151; Figure 2), we show that species scored using a combination of social and genetic mating system generally have larger residual testes mass, steeper Bateman gradients, and more opportunity for sexual selection, all of which are well-established methods of quantifying different aspects of sexual selection. We now add further detail to our justification of EPP as a driver or correlate of sexual selection (lines 99-101), and include an additional citation:**

**Møller, A.P. & Ninni, P. (1998). Sperm competition and sexual selection: a meta-analysis of paternity studies of birds. Behav. Ecol. Sociobiol., 43, 345–358.**

**In the meta-analysis by Møller & Ninni (1998), a key result is that males with more extravagant secondary sexual characters had higher paternity in their own nest compared to less ornamented males, suggesting sexual selection is indeed linked to extra-pair paternity across multiple avian species.**

101 drivers? or correlates? there is a big difference here as correlates are relatively easy to determine from the sorts of patterns you are looking for. To know anything at all about drivers, experimental studies are needed.

**Response: We understand that experimental biologists baulk at the word “drivers” when applied to macroecological studies, and we acknowledge that the results of such studies indicate correlation rather than causation. However, we acknowledge this fact multiple times in the manuscript and in this case the use of the word drivers is used as a general term. The correlates we show are not conclusive but they can provide information about likely drivers (evidence for and against proposed drivers). So we retain the word drivers in this context as we think it is accurately used.**

119 while I appreciate the value in providing the raw data for your study, I cannot evaluate the accuracy of your scoring methods unless you make these data available to reviewers. You also make a big deal, in your closing paragraph, of providing this amazing dataset for others to build/work on, so it seems especially odd that you would want to hide this seemingly important aspect of your research from reviewers. Any fear that you have that reviewers might steal your data is unnecessarily paranoid in my opinion, but one option would be to provide data on a random selection of half or even a quarter of the species.

I often find obvious errors in large datasets like this when checking the scores for the few hundred species that I know reasonably well. Without seeing those data I cannot comment on the validity of your scores and data. Even with the data you provided on 10 species, I can see a few potential errors, and some variables that are not clearly explained.

**Response: We have included an initial dataset example for submission. If reviewers would find it helpful, we could supply the dataset on request.**

129 this is the first time you mention data certainty. How was this scored and used?

**Response: This is a good point as the methods for this part of the study moved to an online section. We now introduce data certainty in greater detail before this point in the ms (Line 133-135). We also reference Table S2 with clear definitions of our scoring criteria for data certainty.**

130 I do not have any confidence in this estimate as I expect that those species with ‘high certainty’ are not a random subset of the full dataset. For example, when DNA fingerprinting first revealed that many species had moderate to high levels of EPP, it became fashionable to publish on species without any obvious EPP whereas publication of yet another species showing moderate levels of EPP became of little interest.

**Response: We agree that our high certainty data subset is a non-random sample of all birds. However, the reviewer seems to have jumped to the conclusion that we mean “genetic evidence”. This is not the case because many high certainty data points are not based on genetic evidence. For example, lekking species (scoring 4) or long-lived socially monogamous seabirds with low extra-pair copulations (scoring 0) can be assigned a score with high certainty through clear-cut behavioural observations. We now clarify this point in the supplementary information (Table S1).**

**The reviewer also seems to have misinterpreted the rationale for the high certainty data. When we analysed geographical variation in data certainty (reported in Figure 3c-d) we found a clear latitudinal gradient, making it very important to account for this gradient when testing for similar latitudinal gradients in sexual selection scores. In all sensitivity analyses, the patterns detected at global scales were maintained in the high-certainty subset, suggesting that biases in knowledge or lack-of-knowledge were not driving our results. Thus the reviewers argument about the so-called “file-drawer” problem of unpublished studies is accurate yet irrelevant.**

131-132 this implies (to me) that you think that species without EPP or biased OSR are not subject to sexual selection

**Response: This is a fair point. We have changed our wording, removing “stronger sexual selection” with “sexual selection via EPP”. We have removed OSR from our scores to simplify the system (a minor change as OSR only contributed to a few species due to low data availability).**

135-145 I do not find this to be at all compelling as a demonstration that your sexual selection scores are valid. One problem is that the dataset used to calculate residual testis size is vastly different from the datasets used to calculate Bateman gradients and Is, in both size and the species involved. The testis dataset is large and represents a large range of species ecologies and behaviours, whereas the Bateman and Is datasets are much smaller and far from a random sample of species. As you indicate, the testis size data reflect post-copulatory sexual selection (sperm competition) whereas the Bateman and Is data are potentially more useful as an overall index of the strength of sexual selection. Note also that the relationship between your scores and Is is decidedly non-linear (Extended Data Fig 2), with the high R2 value being driven by the almost complete lack of variation in your scores for Is>1.0. Thus your claim of an almost perfect fit between Is and your scores is misleading and ignores the fact that your scores are meaningless at accounting for variation in Is>1. I think it is wise and useful to present these analyses but I would suggest a lot more caution in their interpretation.

**Response: We acknowledge that our scores cannot differentiate between species with very high Is, and have toned down our interpretation of the strength of model fit (line 150).**

**This again misses the point because very high Is is found only in a tiny minority of species in a global dataset. Moreover, the patterns suggest that our scores work very well comparing these species with less polygamous species, which was the aim of our study. In future, it may be possible to introduce even higher scores to differentiate the most polygamous lekking species. However, we do not believe that this is (1) currently possible or (2) necessary.**

147 while you do explain what you mean by ‘geographical range data’ in your Methods, a brief description here would really help the reader to understand these patterns.

**Response: We have re-written this paragraph to introduce the concepts and methods needed to understand our mapping of average sexual selection scores worldwide (lines 153-156).**

160 without being able to see you data and certainty scores, I am skeptical of this.

**Response: We included a subset of data with our submission. If it is helpful, we can share a larger section of our data during review.**

**The scoring system for data certainty that we use is exactly the same as in a series of previous papers. In this case, high-certainty sexual selection scores (scored 3-4 for data quality) are available for around a quarter of the world’s birds (n = 2851 species). Our results remain consistent when we restrict our analyses to this better-known subset. This is a standard approach in macroecology to assess whether results can be attributed to biases in knowledge.**

234 ‘became even stronger’ is too strong a conclusion here as there is no evidence for any difference between these two R2 values and the difference is trivial

**Response: This is a fair criticism of our presentation of this analysis. For this reason, we chose to report marginal R2s instead of full R2s, so that phylogeny was not a factor in explaining variation in our scores. This meant the difference between Bateman gradients and opportunity for sexual selection (Is) is now significant. We have also edited our wording to remove “even stronger”, and replaced our wording of Is as “very strong”.**

238-239 this is not correct. Sexual selection is not SUBJECT TO opposing patterns. Rather your analysis suggests different patterns for species with different ecologies.

**Response: Agreed. We have now changed the wording to “sexual selection is subject to opposing latitudinal patterns *in species with different ecologies*”**

241-250 these are probably the most important conclusions from your study

**Response: We agree and have tried to emphasise them and clarify them further in a new version of the text (lines 249-261).**

253-255 shaped by or correlated with. Your study cannot distinguish between cause and effect.

**Response: Experimental biologists are correct to point out that comparative analyses will not conclusively identify causation, only correlation. However, the correlations can give a lot of information about the likely causation, and certainly can reject causal hypotheses. Therefore, it is not correct to strip away all reference to drivers or causes. In this instance, we agree that direction of causality is not confirmed so we changed the wording to “explained by” rather than “shaped by” (line 264). We note that “variation explained by” is common wording when referring to linear models in comparative studies.**

258-259 reference? Simply stating this does not make it true.

**Response: We considered the point to be fairly self-evident, but we agree that a reference would be good to include for clarity. We have now added a citation (Ghalambor et al. 2006) which shows the latitudinal gradient in seasonality very clearly.**

268 or monogamous species tend to evolve territoriality?

**Response: In our view, the evidence for the reviewer’s suggestion is weak but we can see that the direction of causality is debatable so we have changed our wording to “co-evolve” (line 280). This wording highlights the fact that, in the context of our study, it does not matter whether territoriality leads to monogamy or vice versa.**

293, 377 latitudinal gradients??

**Response: We added in “global-scale” gradients to clarify we were referring to latitudinal gradients in space.**

409 well-established?? by whom? reference??

**Response: Our scores are based on thresholds proposed or applied by highly cited works. To be clarify, we edited the text to the following: “Allocation to the first four categories was based on thresholds for polygamy suggested by previous studies: <0.1%, 0.1–5%, 5–20% and >20% for scores of 0–3, respectively (Verner & Willson 1966; Møller 1986; Owens & Hartley 1998; Dale et al. 2015).”**

422 reference??

**Response: We included three new references to illustrate that mating variance is often exceptionally high in lekking species.**

**Mackenzie, A., Reynolds, J.D., Brown, V.J. & Sutherland, W.J. (1995). Variation in male mating success on leks. Am. Nat., 145, 633–652**

**McDonald, D.B. (1989). Correlates of male mating success in a lekking bird with male-male cooperation. Anim. Behav., 37, 1007–1022.**

**Wiley, R.H. (1973). Territoriality and non-random mating in Sage Grouse, *Centrocercus urophasianus*. Anim. Behav. Monogr., 6, 85–169.**

590 link does not work

**Response: The link to our github for code sharing has been set to private and will be changed to public upon acceptance. If the code is necessary for the review process, we are happy to provide a private link to the repository.**

Fig 1 caption: latitudinal gradients?

**Response: We have added the word “latitudinal” to the figure title to make our caption clearer.**

922 which aspect of sexual selection?

**Response: We have included in figure 3 and 4 a brief description of our sexual selection scores, with a reference to Table 1 for specific detailed criteria.**

Fig 4b-e I do not understand these graphs as they appear to show patterns that are far from statistically significant yet they report p-values <0.01. It is quite possible that I am not understanding something here but, if that is the case, I expect that most other readers will have the same problem, so some further clarification is really needed. I assume, for example, that the whiskers and shading show credible intervals (not confidence intervals, as claimed), and that these are the limits derived from the respective Bayesian models. In b,c, and d the two sets of limits overlap, and in e a horizontal (no effect) line is within the shaded region. Thus I am inclined to each of those graphs as showing no evidence for a pattern despite the accompanying p-values. In each case the plausible values of effects for one main effect include plausible values for the other main effect. Please provide an explanation for these apparent anomalies. Also, to my eye, the pattern in 4e is negative and not positive. It is also clear from these graphs that there is a huge amount of variation in each of the categories compared. That suggests to me that, while the differences are apparently statistically significant (if that is indeed the case), the amount of variation explained by the models is small at best. To me that suggests a lot of unexplained variation not accounted for by the models and any claim that these are ‘drivers’ of sexual selection is overblown. At the very least I would like to see your statistical tables include some measure of the amount of variation explained by the models.

**Response: The reviewer is correct that we were referring to “credible intervals” (not confidence intervals). We have corrected this mistake. We also agree that the previous version of the figure was a bit hard to interpret, and that it seemed like the patterns were not supporting our conclusions. The reason for this is partly to do with the method used to generate predicted values for each ecological trait comparison. Initially, we used a standard method that included phylogenetic information to generate predictions, summing the errors for each individual species to generate a single credible interval. We felt this was the most conservative method. However, it meant that our reported statistics, underlying data, and overlying model predictions now seemed to contradict each other.
We have now redesigned the figure for clarity. In new versions of panels b-e, we calculate predicted sexual selection for all species using fixed effects, and the residual error for a typical species (rather than summing errors for all species). This does not affect mean model predictions, but reduces the uncertainty in predictions, so that reported p-values now match the model prediction outputs and better reflect the underlying data. This helps to show that where evidence (and statistical power) is strongest, we show patterns aligned with our stated results and conclusions. We have also expanded the figure caption to explicitly state that our model predictions come from species-level models.**

**Furthermore, in response to the suggestion to report the variation explained by each model, we have now included an additional column in supplementary tables S5 and S6 so that readers can critically assess the importance of each model. We additionally report the explained variance from the multivariate phylogenetic model presented in figure 6. The combination of predictors in the multivariate model explains 45% of variation, with an additional 45% explained by phylogenetic information, suggesting that our models are able to explain a significant proportion of the variance in sexual selection strength across species.**

Extended Data Table 1. I do not agree with some of the criteria here. I fully appreciate that opinions will differ on these but in the face of such differences I think it is important to re-run analyses using different sets of criteria to ensure that your results are robust to different definitions. For example, I would have classified species with occasional or anecdotal records of polygamy as 0 and not 1. The 25+ cases of occasional and anecdotal instances of polygamy that I am aware of are all due to some odd accident or anomaly and cannot be considered important with respect to sexual selection. Moreover, some of the lekking species that I have studied are no more polygamous than your category 3, and certainly would have lower intensity of sexual selection than species with high OSR.

**Response: There will always be disagreement about categories in biology as the boundaries to categories are always fuzzy. However, this is not a problem for our approach as it is assumed in macroecology that categorisations are not watertight. They don’t need to be 100% accurate, but they should be as accurate as possible, with the vast majority of cases falling within broad classification bands. We have worked on this type of classification system for decades, applied to species limits, IUCN threat status criteria, and many life history traits. Here we apply the approach to sexual selection criteria and while we are aware that arguments can be made about whether species A or B is correctly assigned, the overall classification system is nonetheless indicative and useful. The fact that the system contains useful information and is correlated with the intensity of sexual selection is validated in our comparison with other quantitative estimates of sexual selection (see Fig 2).**

**In the context of the specific points made by the reviewer, we disagree that lekking species do not warrant a higher sexual selection score. Lekkers very often have higher rates of polygamy or reproductive skew than non-lekkers and it doesn’t matter if this point applies to <100% of lekkers. To support our argument, we have edited the figure caption: “Our rationale for treating lekking as the most extreme form of polygyny is based on genetic evidence of very high variance in mating success in most of the well-known cases (Mackenzie et al. 1995; McDonald 1989; Wiley 1973; Fig. 2).” The fact that the reviewer knows of cases that appear not to fit is irrelevant to macroecological approaches – and statistical analyses – if the majority of cases fit.**

**To clarify the text, we have removed the word “anecdotal” and replaced with “occasional”. We agree that it may have introduced confusion, as most species scoring 1 for sexual selection are based on consistent but occasional evidence of polygamy, rather than one-off events.**

Extended Data Table 2. In your category 2, you seem to include species with high certainty but variable mating systems. In at least one species that I have studied, we have perfectly good knowledge (i.e., high certainty) of OSR, EPP, and polygamy but those indices change with population density. Your category 3 also seems highly subjective to me and I would want to have this scored by a few knowledgeable ornithologists then each set of classifications tested.

**Response: The reviewer has misunderstood the criterion for category 2 but we can see that this confusion is caused by poor wording on our part. We did not mean to suggest that species with variable mating systems would have lower data certainty scores. The variability we are referring to is when polygamous behaviour is inconsistent across closely related species. This reduces confidence in classification based on inference for a particularly poorly member of the same clade. We have now updated our wording in the classification table (Table S1) to be more explicit:**

**“This includes cases where inference is complicated by heterogeneity of sexual selection scores among closely related species (often caused by high levels of variation in mating systems across a clade) or where published evidence about mating systems is contradictory.”**

**Regarding the subjectivity of the assignments: this is always assumed with this type of scoring system. The key point is that for such broad bands or thresholds, the vast majority of cases are clearly or unambiguously placed. It doesn’t matter that a few cases are misassigned if the scores are strongly correlated with data certainty.**

Extended Data Fig 2. The symbols on these graphs vary in size, which I take to mean that the sizes represent sample sizes of overlapping data. However, these sizes are not explained and I see only one size of data point in ‘a’, which strikes me as odd. Also in ‘b’, you show two dot sizes which I assume represent 1 or 2 species. Yet, if that is true, the graph shows 16 species and not 14. In ‘c’ I those different data point sizes clearly do not represent one or two species. Please provide clarification in the caption, and fix what seem to be errors.

**Response: We now state in the caption “In (b) and (c), point size is scaled by the number of individuals used to calculate sex-specific metrics.”, which we believe is clear and concise.**

Reviewer #3 (Remarks to the Author):

Thank you for the opportunity to review NCOMMS-23-41241-T Resolving the climatic and ecological drivers of geographical gradients in avian sexual selection by Barber et al. In the manuscript, the authors gathered estimates of extra pair paternity and other metrics of sexual selection and used phylogenetic models to disentangle competing hypotheses about global patterns of sexual selection in birds. Results showed that generally sexual selection increases with absolute latitude, and covaries with territoriality, seasonality, and trophic level. Another key finding is that the patterns are reversed in frugivorous birds (highest sexual selection strength in the tropics). Previous work on this topic based on a subset of species found conflicting results, so Barber et al.'s holistic approach is certainly an important contribution to the literature. Additionally, the data from this paper will undoubtedly be used widely by researchers to answer different questions about sexual selection patterns. Thus, this paper will be of interest to the readers of NCOMMs.

I enjoyed reading the paper, and have mostly minor comments for the authors to consider.

**Response: We thank the reviewer for their positive appraisal.**

I do have two main comments: The first is regarding the addition of model selection, as some of the relationships appear non-linear, but they are presented as linear (see comment below). The second is, to the best of my knowledge, the manuscript does not describe how or why data were averaged in 5-degree bins or why data above 70-degrees were not plotted (see comment below).

Abstract
I think the effect of territoriality deserves more focus in the abstract, as it was the strongest effect (Fig. 5).

**Response: We chose to focus on both climate and territoriality, as both had comparable effect sizes.**

Line 20: I have a similar comment below, but because the analyses are based on the Jetz phylogeny, perhaps the authors should state that sample size too.

**Response: Although our analyses were conducted using the Jetz phylogeny, which has been a fantastic resource for macroecologists for over a decade, the taxonomy of the tree (and accuracy of taxonomically placed branches) is becoming increasingly obsolete.**

**We therefore prefer to report the sample size for the Clements dataset in the abstract. This is the more recent and accurate taxonomy, and the forthcoming Clements-based birdtree will be the main phylogeny used in future research in avian macroevolution. Therefore, we want to make sure our sexual selection data is aligned for immediate use with the forthcoming tree, and to highlight the importance of the dataset for future research. We have edited the text to clarify that the main dataset is Clements, and that we also provide and use the data in Jetz format. We have tried to clarify that both these datasets contain “all birds” just with different species limits.**

Introduction
Line 48 onwards: The rationale for the competing hypotheses presented here have long been difficult for me to understand (indeed the main reason I think this is an important study). For example, the high-latitude hypothesis relies on synchronous breeding to explain strong sexual selection, while the tropics implicates asynchronous breeding. The authors go on to explain methodological issues that may have produced conflicting results, but I was hoping for more text here on the mechanisms driving the selection pressures such as the role of diet and territoriality. I think some more discussion on tropic level and territoriality effects would also serve to set-up those results too. Could the authors expand on this a bit more here?

**Response: We are glad the reviewer agrees that the question is a thorny one, and worth exploring. We would like to include more background information in the introduction but we feel this overcomplicates this section. Instead, we have added a more in-depth review on the roles of diet and territoriality to the discussion.**

Line 61: I might refer to the hypotheses as "competing," because they do seem to be in conflict.

**Response: We chose to retain the term “alternative hypotheses”, as our results have shown that both positive and negative latitudinal gradients in sexual selection exists, depending on ecological context.**

Results
Some relationships appear to be non-linear but are presented as linear (e.g., Extended Data Fig. 3D looks quadratic). Can the authors present some model selection results indicating the relationships are linear? Further, a key point in the discussion references Extended Data Fig. 8 (lines 282-297). Are the plotted results in panel B correct? The relationship looks negative.

**Response:**

**The curved lines visible in some figures reflect the use of ordinal models, which transform responses to the log-scale (i.e. like normal logistic regression). Since we are showing model predictions on the linear scale, they are curved. The reviewer may be misinterpreting these curves as non-linear responses. We prefer not to introduce complex quadratic curves because (1) it is not standard procedure in macroecological analyses, and (2) it would add a lot of analytical complexity and look like “data dredging”.**

**As for the comment about relationships on figures that seem to differ from what we report, the reviewer raises an important point. We acknowledge that the style of visual presentation we used in the last version of the ms was misleading, with the underlying plotted data sometimes contrasting with the model predictions and reported statistics. This is mainly due to unequal sample sizes for each latitudinal partition, which were not shown. For example, despite higher latitudinal bins containing very few species and highly uncertain average scores, the plots made them appear like they were contributing equally to our analyses, whereas most of the statistical power for the analyses was derived from bins at lower latitudes containing far more species.**

**To address this issue, we made changes to each plot containing latitudinal gradients. (1) We now remove any latitudinal bin with < 10 species, consistent with removing grid cells with < 10 species from maps. (2) We have resized the data points to scale with sample size, which reveals the unequal sampling across the latitudinal gradient. These edits show where the evidence (and statistical power) is strongest, so that the plots match our stated results and conclusions.**

Discussion
Line 238: Should this read "nearly all" rather than "all birds"? The authors note there is disagreement between the Jetz and Clements phylogenies and the analyses were based on the Jetz phylogeny that has fewer species.

**Response: The reviewer seems to have misunderstood the implications of shifting taxonomy. The Jetz list has 1000+ species fewer than the Clements list and yet both are lists of “all birds” (just one splits them up differently). Nonetheless we can see that readers may be confused by the different species totals so to side-step the issue we have changed the wording to “Based on a newly compiled global dataset” (Line 249).**

Lines 282-297: This section is a little confusing to me, and I suspect it will be of much interest to readers, so I am asking the authors to clarify it. The authors state climate stability in the tropics free birds from territorial resource defense (lines 286-291), and in lines 291-297 the authors state stable climates promote territoriality. However the relationship in Extended Data Fig. 8 all look negative. Perhaps the source of my confusion is from Extended Data Fig. 8B. Are the plotted data correct? That relationship looks negative, which would mean higher territoriality in the tropics for primary consumers. Can the authors please clarify these results?

**Response: We acknowledge that our ED figure 8 (now figure S9) may have been confusing to readers, as the data points appeared to show a trend inconsistent with the trend line generated from model predictions.**

**The issue in question was the same issue raised by reviewer #3 regarding figure 3. The changes we made (remove < 10 species data points and resizing points) were applied to all figures with maps and latitudinal gradient. Consequently, figure S9 data points now show a positive trend, consistent with the overlaid model predictions.**

Methods
What is the rationale for averaging sexual selection scores into 5-degree latitudinal bins? The error bars suggest there is a lot of variation, so I would like to see the raw data plotted or at least some explanation for the 5-degree binning.

**Response: We found initially plotting 10,000 species as individual points greatly reduced the ability to determine any global trends, due to overplotting discrete variables on a continuous scale.**

**To address this issue, we opted to use mean values and 95% confidence intervals for each 5 degree latitudinal bin. Note that plotting latitudinal data in bins for ease of interpretation is common practice in macroecology. To clarify our approach, we have updated all relevant figure captions with: “variables were averaged within five-degree latitudinal bins to reduce overplotting”.**

Figures
Data above 70-degrees latitude are not plotted, why?

**Response: We removed points above 70 degree latitude because they were based on very few species (<10), and there was high uncertainty in mean values. As mentioned above, we have now removed all latitudinal bins with fewer than 10 species, regardless of their location. We prefer this approach because it is more standardised, easier to explain and also means that the same threshold (<10) is used for both producing maps and visualising latitudinal trends. Note that discarding grid cells and bins with averages calculated from very low numbers of species is common practice in macroecological studies to avoid chaotic plots. We have updated all relevant figure captions to say: “To reduce noise, cells and latitudinal bins with <10 species were excluded from all plots.”**

I suggest adding "breeding latitude" to all relevant figure captions.

**Response: Agreed. We have now added “breeding latitude” to all relevant figure captions to be more explicit.**

Supplementary material
L 209: References Fig S2 for posterior predictive checks but Fig S2 is the distribution of high certainty data. Is this correct?

**Response: Thanks for pointing out this error. Our posterior predictive checks were only included in a preliminary draft and were removed because we felt the graph would be confusing to the majority of readers, except those with extensive experience in both Bayesian and ordinal models.**
