# Supplementary material for: Climate and ecology predict latitudinal trends in sexual selection inferred from avian mating systems
Source: PLoS Biol. 2024 Nov 4;22(11):e3002856. doi: 10.1371/journal.pbio.3002856 (PMC11567637; doi:10.1371/journal.pbio.3002856)
Supplement: Attachment — Submitted filename: Responses 29 Aug.docx [file pbio.3002856.s017.docx]

COMMENT

Please could you change your Title very slightly to "Climate and ecology predict latitudinal trends in sexual selection inferred from mating systems in birds"?

RESPONSE

We have discussed this request with the editor, who agreed to retain the original title on the grounds that it is more succinct and flows better, and that “avian” is a commonly used term that will be understood by a broad readership.

COMMENT
Please attend to the remaining request from reviewer #2. The Academic Editor notes that you may already have attempted to do this in the previous round, so this is not a hardline requirement, but anything you can do in this direction, bearing in mind our broader readership, would be helpful.

“Reviewer #2: I have only read briefly through the revised manuscript and the authors replies, but it is clear that authors have genuinely taken on board my original concerns with some aspects of their analyses, and gone to considerable lengths to carry out new analyses of their data to address these. The result is now a far more robust set of results that more strongly support their conclusions. I still think that the conclusion could do with a more clear take-home message or statement of the broader implications of the findings.”

RESPONSE

We thank the reviewer for their positive appraisal.

We agree that our attempts to clarify the take-home message in the conclusions may have fallen a bit short, so we have made substantial further edits to the concluding paragraph. We hope this now does a better job of conveying the main findings and their wider importance.
